# Identification of miR-6794-3p as a suppressor in pancreatic cancer metastasis

**DOI:** 10.7150/ijbs.98490

**Published:** 2024-09-30

**Authors:** Ha Gyeong Kim, Yunmi Cho, Jae-Seon Lee, Eun-Taex Oh, Heon Joo Park

**Affiliations:** 1 Department of Microbiology, College of Medicine, Inha University, Incheon 22212, Republic of Korea.; 2 Program in Biomedical Science & Engineering, Inha University, Incheon 22212, Republic of Korea.; 3 Department of Molecular Medicine, College of Medicine, Inha University, Incheon 22212, Republic of Korea.; 4 Research Center for Controlling Intracellular Communication, College of Medicine, Inha University, Incheon 22212, Republic of Korea.; 5 Department of Biomedical Sciences, College of Medicine, Inha University, Incheon 22212, Republic of Korea.

**Keywords:** miR-6794-3p, pancreatic cancer, metastasis, RBBP4, GRHL2

## Abstract

Metastasis is a major cause of treatment failure in patients with pancreatic cancer, highlighting the urgent need for effective therapeutic strategies. Here, we focused on identifying novel miRNAs with key roles in metastasis of pancreatic cancer. Microarray analysis of miRNA expression in metastatic and non-metastatic pancreatic cancer samples revealed significantly lower expression of miR-6794-3p in the metastatic tumor group. Gain- and loss-of-function approaches using the pancreatic cancer cell lines MIA-PaCa-2 and HPAF-II expressing low and high levels of miR-6794-3p, respectively, indicated a role of miR-6794-3p in suppression of cell invasion, migration, and EMT signaling. Importantly, our results showed that miR-6794-3p exerts its effects by inhibiting expression of the chromatin remodeling factor, RBBP4. The resulting suppression of RBBP4 induced an increase in the levels of GRHL2 involved in regulating invasion, migration, and EMT signaling in metastatic pancreatic cancer cells. Consistent with these findings, low miR-6794-3p expression levels correlate with poor pancreatic cancer patient survival. Additional preclinical experiments on nude mice clearly demonstrated inhibitory effects of miR-6794-3p on pancreatic cancer cell metastasis. The collective results highlight the functional significance of miR-6794-3p as a suppressor of metastasis and support its predictive utility as a prognostic biomarker and therapeutic target in pancreatic cancer.

## Introduction

Incurable pancreatic ductal adenocarcinoma (PDAC) is the most common type of pancreatic cancer, with overall survival rates increasing only slightly from 3% to 9% over the past 50 years 1. This modest improvement remains significantly lower than that of many other tumor types, highlighting the clinical importance of effective management of pancreatic cancer 2,3. Treatment strategies for pancreatic cancer vary widely depending on tumor stage. Although adjuvant therapy has been shown to improve prognosis after surgery, tumors are resectable in a low proportion of patients 4,5. While combination chemotherapy is a common option, the therapeutic efficacy is limited in metastatic cases 6. Therefore, the development of methods that can predict the likelihood of metastasis at the time of diagnosis and further impede tumor progression is a crucial unmet medical need.

PDAC is characterized by several germline or acquired genetic mutations, the most common being *SMAD4*, *TP53* and *KRAS* 7,8. There are anticancer drugs that target these genes 7,8, and additional research is needed to find effective anticancer drugs. Recently, miRNAs have attracted substantial attention as prospective targets in cancer diagnosis and therapeutics due to their association with tumor progression 9. As post-transcriptional regulators, miRNAs function as either oncogenic or tumor suppressor genes via direct binding to the 3'-UTR region of target messenger ribonucleic acids and repressing their expression through degradation or translational inhibition 9. In many tumor types, more than half of the miRNAs are abnormally expressed 8-10. These findings have further fueled research interest in characterizing the functions of miRNAs expressed in pancreatic cancer cells for potential application in treatment and diagnosis.

Multiple studies have examined the involvement of miRNAs in pancreatic cancer 9. Serum carbohydrate antigen 19-9 (CA19-9) is commonly used as a marker for assessing clinical treatment efficacy in pancreatic cancer 11. While CA19-9 remains the only FDA-approved marker for disease management, its use is associated with several limitations, including ineffectiveness, low sensitivity, and low specificity 11. Earlier studies indicate that miRNA-483-3p and miRNA-21 have potential utility as biomarkers of PDAC from blood plasma 12. Comparative analyses of miRNAs between pancreatic cancer and normal pancreas tissues have been conducted 13. In pancreatic cancer tissues, miRNA-424, miRNA-100, miRNA-301, miRNA-212 and miRNA-125b-1 are reported to be overexpressed while miRNA-345, miRNA-142-P and miRNA-139 levels are decreased relative to normal tissues 13. Furthermore, increased expression of miRNA-155 and miRNA-21 in precursor lesions is documented, implying that miRNA-155 could serve as a specific biomarker in pancreatic juice 14. Recent studies have focused on miRNAs associated with metastasis in pancreatic cancer. Several miRNAs, including miR-301a, miRNA-148a, miR-186, and miRNA-326, are implicated in the invasion and metastasis processes 15-17. The majority of studies to date have investigated changes in expression and function of miRNAs between normal and pancreatic cancer tissues. To identify the miRNAs specifically associated with metastasis and invasion, metastatic and non-metastatic tissues of pancreatic cancer were evaluated in the current study.

Nuclear retinoblastoma binding protein 4 (RBBP4) regulates the transcription of numerous genes 18. RBBP4 is a component of several chromatin modification structures, including as polycomb repressor complex 2 (PRC2), deacetylation and nucleosome remodeling complex 19, and SIN3-chromatin modulating complex 20. The involvement of RBBP4 in the development of various tumor types is well established 21. Aberrant expression of RBBP4 is implicated in poor prognosis and metastasis of multiple highly metastatic and invasive tumors, including colon cancer 21, neuroblastoma 22, and lung cancer 23. Furthermore, high expression of RBBP4 is proposed to contribute to the poor outcomes of breast cancer therapy 24. However, the specific role of RBBP4 in pancreatic cancer cell invasion and migration is yet to be elucidated. Previous reports have shown that miR-145 and miR-885-5p target RBBP4 to regulate non-small cell lung cancer cell proliferation 25 and glioblastoma carcinogenesis 26, respectively. In addition, miR-429 is reported to promote liver tumor-initiating cell properties through targeting of RBBP4 27. However, to our knowledge, no RBBP4-targeting miRNAs involved in tumor cell invasion and migration have been identified in pancreatic cancer.

The Grh (Grainyhead) gene was originally identified in *Drosophila*, following which three mammalian homologs were reported (GRHL1, GRHL2 and GRHL3) 28-29. GRHLs regulate the expression of genes encoding epithelial cell-cell junction proteins, cytoskeletal regulation, membrane trafficking, and guidance cues 29. GRHL2 is upregulated in many cancer types, including colorectal, breast and oral squamous cell carcinoma 29, and can act as an activator or suppressor of target gene transcription by interacting with promoter and enhancer regions in cooperation or competition with other transcription factors and epigenetic regulators 29. GRHL2 is reported to suppress aspects of tumor progression through inhibition of EMT 29 and downregulated at the invasive front of breast cancers. Furthermore, loss of GRHL2 expression in primary breast cancers is correlated with lymph node metastasis 30. Silencing of *GRHL2* induces EMT in lung cancer cell lines with distinct effects on proliferation and clonogenic growth 31. The collective results suggest that GRHL2 may exert its effects in a tumor type- and stage-specific manner through regulating different target genes in multiple cancer types 32. GRHL2 is more strongly expressed in normal pancreatic duct than invasive pancreatic ductal cells and regulates epithelial plasticity along with stemness in PDAC, both of which are crucial for metastasis 33. However, in pancreatic cancer, the miRNAs that regulate GRHL2 and, in turn, tumor cell invasion and migration are currently unknown.

In this study, microarray analysis of miRNA expression patterns in metastatic and non-metastatic pancreatic cancer specimens revealed markedly lower miR-6794-3p expression in metastatic tissues. We further explored the factors underlying decreased miR-6794-3p expression in metastatic pancreatic cancer and its potential roles in tumorigenesis. Our collective findings suggest that miR-6794-3p regulates invasion and migration of pancreatic cancer cells through targeting the RBBP4/GRHL2 signaling pathway. Furthermore, low-level expression of miR-6794-3p was consistently correlated with poor patient survival.

## Materials and Methods

### Patients and specimens

Written informed consent approved by the Institutional Review Board of Inha University Hospital Clinical Trial Center (IRB No. 2017-07-012; Incheon, Republic of Korea) was obtained from all the patients who were to undergo metastatic- (n=5) and non-metastatic- (n=5) tumor tissues for pancreatic cancer. All tissues were frozen in liquid nitrogen and stored at -80 °C.

### Pancreatic cancer cell lines and culture conditions

Human PDAC cells including MIA-PaCa-2, HPAF-II, Panc-1, AsPC-1, and BxPC-3, were purchased from American Type Culture Collection (ATCC, USA) and cultured in Dulbecco's modified Eagle's medium (DMEM) Minimum Essential medium (MEM) or RPMI 1640 medium. PATU-8988T and PATU-8988S were obtained from Deutsche Sammlung von Mikroorganismen und Zellkulturen (DSMZ, Germany). The cells were cultured in MEM. Cells were incubated at 37 C in a 5% CO2-containing humidified incubator unless otherwise noted.

### Chemicals and antibodies

Thymidine, 5-Aza-2`-deoxycytidine (5-Aza), β-glycerophosphatate, sodium orthovanadate, and sodium fluoride, and GSK126 were acquired from Sigma-Aldrich, USA, along with turbofect reagent and lipofectamine reagent were obtained from Thermo Fisher Scientific (USA). Antibodies were obtained from the following companies: anti-Cytokeratin 8 (#SC-8020, Santa Cruz Biotechnology, USA), anti-Keratin 19 (#4558S, Cell Signaling Technology, USA), anti-β-catenin (#8480S, Cell Signaling Technology, USA), anti-ZEB1 (#70512, Cell Signaling Technology, USA), anti-β-actin (#A1978, Sigma-Aldrich, USA), anti-RBBP4 (#NB100-60399, Invitrogen, USA), anti-GRHL2 (#ab88631, Abcam, USA), anti-Histone H3 (#4499, Cell Signaling Technology, USA), anti-Tri-Methyl-Histone H3 (Lys27) (#9733, Cell Signaling Technology, USA), and anti-Acetyl-Histone H3 (#8173, Cell Signaling Technology, USA). Secondary antibodies were purchased from the following companies: anti-rabbit HRP (#7074, Cell Signaling Technology, USA) and anti-mouse HRP (#7076, Cell Signaling Technology, USA).

### RNA preparation and microarray

Total RNA was extracted from tumor tissues using TRIzol reagent (Invitrogen, USA) in keeping with the manufacturer's instructions. For each sample, 100 ng of RNAs were labeled with a FlashTag Biotin HSR Labeling Kit (Genisphere - Affymetrix UK Ltd, United Kingdom). Samples were hybridized for 16 h at 48 °C on a GeneChip microRNA 4.0 Array (Affymetrix, United Kingdom) and scanned with a GC30007G scanner (Affymetrix, United Kingdom). Raw data were normalized using the Expression Console 1.4.0 (Affymetrix, United Kingdom) with the RMA method, algorithmically based on microarrays spike-in and called to the standard normalization. The differentially expressed miRNAs between metastatic- and non-metastatic- tumor tissues were identified for those that showed more than 1.5-fold changes at a false discovery rate of less than 10%.

### RNA isolation and quantitative polymerase chain reaction (qPCR)

Total RNA was extracted from cancer cells or tissues using the TRIzol reagent (Invitrogen, USA) and treated with DNase I (New England Biolabs, USA). cDNA was synthesized from (1 μg) of total RNA using AccuPower RT PreMix (Bioneer Corporation, Daejeon, Republic of Korea), and then amplified by PCR using the appropriate primer pairs. The primer pairs for *RBBP4, GRHL2, MAST1, KRT8, KRT19*, *CTNNB1*, *ZEB1*, *ATP6AP2*, *BCAT1*, *CA3*, *EPDR1*, *IL7*, *KIAA0087*, *NUDCD1*, *PAX4*, *PON2*, *TLNRD1*, *TMCO1*, *MAST1, VIM, CDH1* and *18s rRNA* were purchased from Bioneer Corporation, Daejeon, Republic of Korea. The primer sequences for the genes are listed in **Supplementary Table 1**. qPCR and analyses were performed using a CFX Connect Real-Time PCR Detection System (Bio-Rad, USA). miRNA first strand was synthesized from (1 μg) of total RNA using Mir-X miRNA First-Strand Synthesis kits (TaKaRa, Japan). The primers for miRNAs were purchased from Qiagen, USA. qPCR was performed using iQ SYBR Green Supermix (2×) (Bio-Rad, USA) and analyzed with the CFX Connect^TM^ Real-Time PCR Detection System (Bio-Rad, USA).

### Boyden chamber invasion and migration assays

Cells were treated with 1 mM thymidine for 24 hr, resuspended in serum-free media and seeded in the upper uncoated chamber for migration assay or upper chamber coated with matrigel (BD Biosciences, USA) for invasion assay, and the media supplemented with 20% FBS was added to the lower chamber. After 24 hr for migration and 48 hr for invasion, the methanol and crystal violet (0.2%) were used to fix and stain the migrated or invaded cells for 15 and 20 min, respectively, and then quantified visually in three random field using bright field optics.

### Nuclear and cytoplasmic protein extraction

To obtain the cytoplasmic fraction, cells were resuspended in 300 μl of Tween 20 lysis buffer (25mM Tris, pH 8.0, 0-50mM NaCl, 2mM EDTA, 1mM phenylmethylsulphonyl fluoride, 0.5% Tween 20). The samples were incubated on ice for 15 min, and the cytoplasmic proteins were harvested by centrifugation at 6,000g for 1 min at 4 °C. The nuclear pellets were resuspended in 100 μl of RIPA buffer containing 500 mM NaCl and incubated on ice for 15 min. The nuclear proteins were harvested by centrifugation at 10,000g for 1min at 4 °C. The samples were assessed by SDS-PAGE and immunoblot analysis. The immunoblot analysis results were determined by chemiluminescence and ChemiDocTM.

### Immunoblot analysis

Cells were lysed using ice-cold RIPA buffer containing a protease inhibitor cocktail (Roche Applied Science, Germany), sodium orthovanadate (Sigma-Aldrich, USA), and sodium fluoride (Sigma-Aldrich, USA). Proteins in whole-cell lysates were resolved by sodium dodecyl sulfate-polyacrylamide gel electrophoresis (SDS-PAGE) and analyzed by immunoblotting. Signals were detected using enhanced chemiluminescence reagents (Thermo Fisher Scientific, USA). Uncropped images of the blots are shown in **Supplementary Figure S9**.

### Transfection of siRNA, miRNA mimics and miRNA inhibitors

RBBP4 and GRHL2 were subjected to RNA interference using a 19 bp (including a 2-deoxynucleotide overhang) small interfering RNA (siRNA). siRNA against *RBBP4* and *GRHL2* were purchased from Bioneer Corporation (Daejeon, Republic of Korea), with negative control RNA (Bioneer, Daejeon, Republic of Korea) used as a negative control. The sequences for siRNAs are listed in **Supplementary Table 2**. miRNA mimics and inhibitors used in this study were purchased from Bioneer Corporation, Daejeon, Republic of Korea. The sequences for miRNA mimics and miRNA inhibitors are listed in **Supplementary Table 2**. Cells were seeded in 25 cm^2^ flasks, grown to ~80% confluence, and transfected with siRNA duplexes using LipofectAMINE 2000 (Invitrogen, USA) according to the manufacturer's protocol. After 48 h, cells were processed for analysis as indicated.

### Reporter assays

Cells (5 × 10^4^) were seeded into 24-well plates, incubated overnight, and co-transfected with 50 μl mixture containing 1 μg p*luc*-*RBBP4*-3'-UTR WT, p*luc*-*RBBP4*-3'-UTR 1∆, p*luc*-*RBBP4*-3'-UTR 2∆, p*luc*-*RBBP4*-3'-UTR 3∆, 3 × GRE or 5 × GRE and 0.01 μg pRL-*luc* (transfection control; Promega, USA) using the TurboFect *in vitro* transfection reagent (Fermentas, USA). After 4 h, cells were washed with PBS and incubated with the appropriate medium for 48 h. Luciferase activity was determined using a luciferase assay system (Promega, USA) and normalized with respect to *Renilla* luciferase activity according to the manufacturer's instructions. Three independent transfections were performed in each case.

### Methylation-specific PCR (MSP)

The sequences of the primers were analyzed via the MethPrimer (https://www.urogene.org/methprimer). MIA PaCa-2 cells (1 × 10^5^) were seeded into T25-cm^2^ flask and treated with 5-Aza for 5 days. Samples were prepared to for MSP using EZ DNA Methylation-Gold Kit (Zymo Research, USA). Methylation of promoter CpG islands were amplified via PCR with the appropriate primer pairs. The primer pairs were purchased from Bioneer Corporation, Daejeon, Republic of Korea. The sequences for primer pairs are listed in **Supplementary Table 3**.

### Construction of plasmids and stable cell lines

To construct plasmids expressing RBBP4 and GRHL2, total RNA was obtained from cancer cells using TRIzol reagent (Invitrogen, USA) and cDNA generated using SuperScriptTM III Reverse Transcriptase (Invitrogen, USA). The open reading frames (ORF) of RBBP4 and GRHL2 were amplified via PCR with the appropriate primers. The primer pairs were purchased from Bioneer Corporation, Daejeon, Republic of Korea. The sequences for primer pairs are listed in **Supplementary Table 4**. Amplified products were digested with restriction enzymes and directly ligated into pCDNA3.1-myc-His_6_ (Invitrogen, USA) vector for cloning. The cloned plasmids were analyzed via restriction digestion and DNA sequencing (Bionics, Seoul, Republic of Korea). To construct luciferase reporter plasmids encoding the wild-type and mutant 3'-UTR of RBBP4, total RNA was obtained from cancer cells using TRIzol reagent (Invitrogen, USA) and cDNA generated using SuperScriptTM III Reverse Transcriptase (Invitrogen, USA). The wild-type and mutant 3'-UTR of *RBBP4* were amplified via PCR with the appropriate primer pairs. The primer pairs were purchased from Bioneer Corporation, Daejeon, Republic of Korea. The sequences for primer pairs are listed in **Supplementary Table 5**. Amplified products were digested with restriction enzymes and directly ligated into pGL-3 control vector (Promega, USA) for cloning. The cloned plasmids were analyzed via restriction digestion and DNA sequencing (Bionics, Seoul, Republic of Korea). To construct luciferase reporter plasmids encoding the 3 × GRE and 5 × GRE, we used nucleotides. The nucleotide pairs were purchased from Bioneer Corporation, Daejeon, Republic of Korea. The sequences for nucleotide pairs are listed in **Supplementary Table 6**. After the hybridization of nucleotides, the double strand DNA of 3 × GRE and 5 × GRE was cloned and directly ligated into pGL-3basic vector (Promega, USA) for cloning. The cloned plasmids were analyzed via DNA sequencing (Bionics Seoul, Republic of Korea). The plasmid expressing MAST1 (#VB900132-7646rqa) was purchased from Vector Builder, USA). The plasmid miR-Cont (#pCMVMIR) and miR-6794-3p mimic (SC402235) were obtained from Origene, USA. The plasmids for miR-Cont (#CmiR-AN0001-SN) and miR-6794-3p inhibitor (#HmiR-AN3685-AM02) were purchased from Gene Copoeia, USA. To construct plasmids expressing exon and intron of MAST1, total DNA was extracted from cancer cells using the AccuPrep® Genomic DNA extraction kit (Bioneer Corporation, Daejeon, Republic of Korea). The exon and intron of MAST1 and pre-miR-6794-3p were amplified via PCR with the appropriate primer pairs. The primer pairs were purchased from Bioneer Corporation, Daejeon, Republic of Korea. The sequences for primer pairs are listed in **Supplementary Table 7**. To construct plasmid shGRHL2, the nucleotide pairs were purchased from Bioneer Corporation, Daejeon, Republic of Korea. The sequences for nucleotide pairs are listed in **Supplementary Table 7**. After the hybridization of nucleotides, the double strand DNA of shGRHL2 was cloned and directly ligated into pshCont vector (Vector Builder, USA) for cloning. The cloned plasmids were analyzed via DNA sequencing (Bionics, Seoul, Republic of Korea). To construct stable cell lines, cells were seeded at a density of 5 × 10^4^ cells per well in 24-well plates and transfected with 50 μl mixture containing 1 μg DNA along with TurboFect *in vitro* transfection reagent (Fermentas, USA). Transfected cells were selected with 1 mg ml^-1^ G418 or 1 μg ml^-1^ puromycin (Duchefa Biochemie, Netherlands) for 1 week and maintained in DMEM or RPMI-1640 containing 0.5 mg ml-1 G418 or 0.3 μg ml^-1^ puromycin during the experiments.

### Animal experiments

The animal experiments in this study were approved by the Animal Care Committee of Woojungbio. Co., LTD, Suwon, Republic of Korea) (IACUC Permit Number: IACUC2023-049). For *in vivo* lung metastasis model, the nude mice were randomly divided into four groups (n = 6), and 2 × 10^6^ cells were injected through the tail vein. Five weeks later, all of the mice were sacrificed, and the lungs removed, paraffin-embedded and stained with hematoxylin and eosin (H&E).

### ChIP assay

ChIP assays were performed using the ChIP Assay Kit (Sigma-Aldrich, USA) according to the manufacturer's protocol. Briefly, cancer cells incubated in a T25-flask were crosslinked by treatment with formaldehyde (final concentration, 1%) for 10 min at room temperature. After washing with PBS, cells were pelleted and resuspended in SDS lysis buffer (1% SDS, 10 mM EDTA, 50 mM Tris-HCI (pH 8.1), 1 mM DTT, and 1 mM PMSF). The lysates were then subjected to sonication to reduce the DNA length to between 500 and 1000 bp, diluted with dilution buffer (0.01% SDS, 1.1% Triton X-100, 1.2 mM EDTA, 16.7 mM Tris-HCI (pH 8.1), 167 mM NaCl), and pre-cleared by incubating with a Salmon Sperm DNA/protein A agarose-50% slurry for 60 min at 4 °C. The supernatant was incubated with anti-RBBP4 (NB100-60399, Novus, USA) or anti-Rabbit normal IgG (#3900, Cell Signaling Technology) at 4 °C overnight. Immunocomplexes were collected with the Salmon Sperm DNA/protein A agarose-50% slurry and eluted after extensive washings, and crosslinking was reversed by heating at 65 °C, followed by treatment with 40 mg/ml proteinase K at 45°C for 60 min. DNA was recovered by phenol-chloroform/ ethanol precipitation, and was used as a template for PCR to amplify the target sites in the *GRHL2* promoter with the following primer pairs: PCR was performed with the following primer pairs: 5'-CCT TCC CAT CCC TGC TTT GGA GAA G-3' (forward) and 5'-CTC CCT CAG GTG AGC TGC CAT TGG CA-3' (reverse). The PCR products were electrophoresed on a 1.5% agarose gel and stained with ethidium bromide (EtBr) (Sigma-Aldrich).”

### Statistical analysis

All error bars represent mean ± SEM. Differences between groups were analyzed with unpaired *t* test. Analysis was performed using GraphPad Prism software (GraphPad, USA). (* *P* < 0.05, ** *P* < 0.01, *** *P* < 0.001, **** *P* < 0.0001, NS indicates no significance).

## Results

### Identification of miR-6794-3p as a potential regulator of pancreatic cancer metastasis

**Figure 1:**
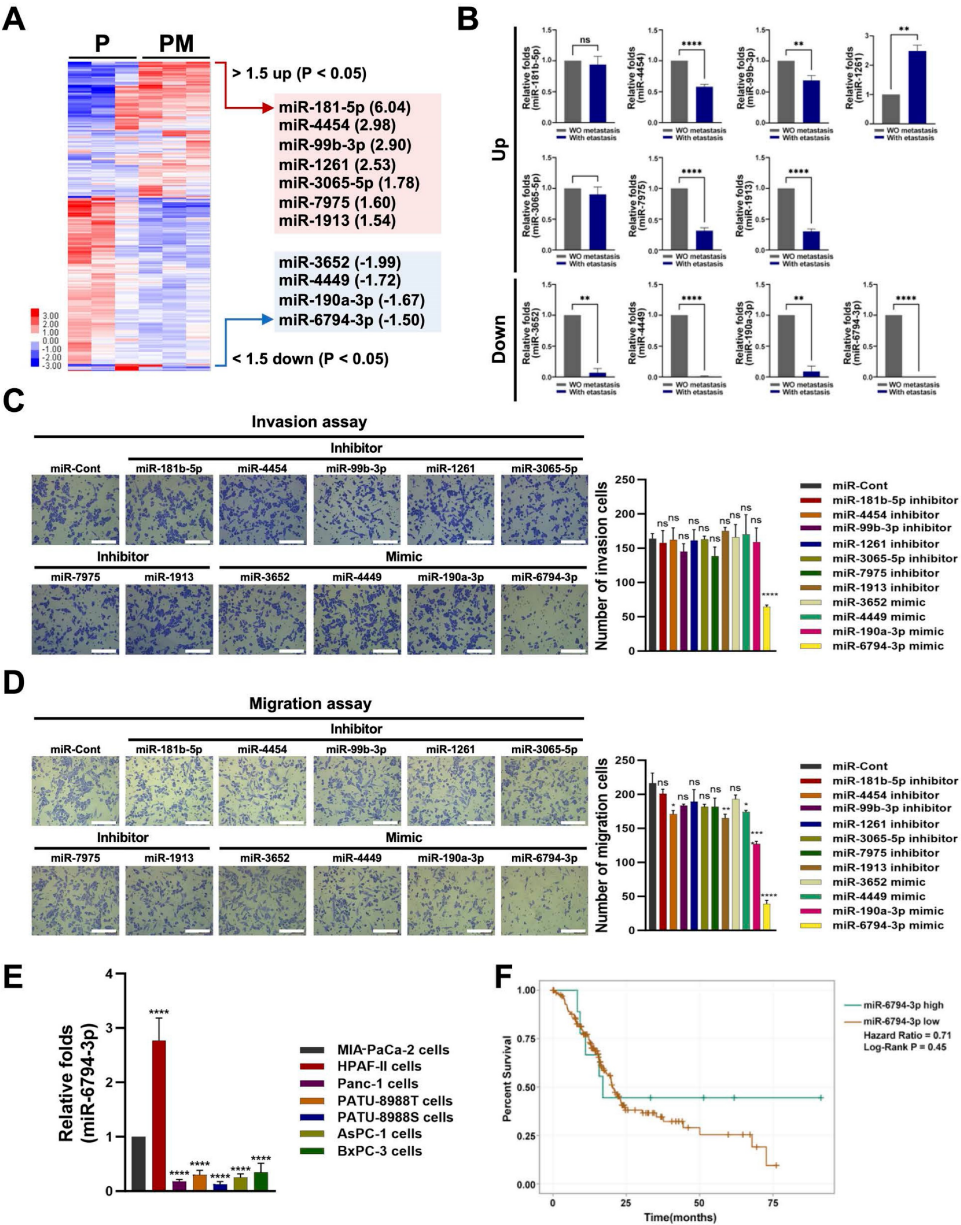
Identification of miR-6794-3p as a potential regulator of pancreatic cancer metastasis.** (A)** Heatmap of the miRNA profiles of metastatic and non-metastatic pancreatic cancer tissues. Red represents high abundance and blue represents low abundance. **(B)** qPCR analysis of expression patterns of selected miRNAs in metastatic and non-metastatic pancreatic cancer tissues. **(C-D)** Invasion **(C)** and migration **(D)** assays of MIA-PaCa-2 cells transfected with mimics or inhibitors of selected miRNAs. Left panel: Representative images of invasion and migration of cells; Right panel: Quantification of invasion and migration. All data are presented as mean ± SEM. * *P* < 0.05 with unpaired *t* test, **** *P* < 0.0001 with unpaired *t* test. Ns represents no significance. Bar = 200 μm **(E)** Relative levels of miR-6794-3p in MIA-PaCa-2, HPAF-II, PANC-1, PATU-8988T, PATU-8988S, AsPC-1 and BxPC-3 cells. qPCR analysis of miR-6794-3p expression was performed using *U6 snRNA* as the internal control. All data are presented as mean ± SEM. *** *P* < 0.001 with unpaired *t* test, **** *P* < 0.0001 with unpaired *t* test. **(F)** Based on miR-6794-3p expression, overall survival of patients with pancreatic cancer was analyzed using the Kaplan-Meier method.

To identify miRNAs that regulate pancreatic cancer metastasis, we performed microarray analysis on metastatic and non-metastatic pancreatic cancer tissues. The expression levels of miR-181-5p, miR-4454, miR-99b-3p, miR-1261, miR-3065-5p, miR-7975 and miR-1913 were increased at least 1.5-fold, while those of miR-3652, miR-4449, miR-190a-3p, and miR-6794-3p were decreased at least 1.5-fold in metastatic pancreatic cancer tissues (PM) compared to non-metastatic pancreatic cancer tissues (P) (**Figure 1A**). It has been reported that microarray observations be verified by quantitative q-PCR or other techniques 34. Using the optimal method, 91 to 95% of the gene expression differences between cell lines identified through cDNA microarray analysis could be confirmed through q-PCR 34. Therefore, we performed qPCR analysis of each miRNA found to differ between metastatic and non-metastatic pancreatic cancer tissues in our microarray analysis. Consistent with the microarray data, the expression level of miR-1261 was increased and those of miR-3652, miR-4449, miR-190a-3p, miR-3652, and miR-6794-3p were decreased in metastatic pancreatic cancer tissues compared to non-metastatic pancreatic cancer tissues (**Figure 1B**). We observed some differences between the microarray and qPCR data (**Figure 1A, B**). Therefore, to confirm the effect of the screened miRNAs on pancreatic cancer metastasis, MIA-PaCa-2 cells (a human pancreatic cancer cell line) were treated with a mimic or inhibitor of each screened miRNA, and invasion and migration analyses were performed. Low levels of miR-6794-3p expression were associated with pancreatic cancer cell invasion, whereas the other miRNAs did not appear to affect pancreatic cancer cell invasion (**Figure 1C**). In migration assays, low levels of miR-190a-3p and miR-6794-3p expression had greater impacts on pancreatic cancer cell migration than the other miRNAs (**Figure 1D**). Since low levels of miR-6794-3p expression impacted both invasion and migration of pancreatic cancer cells in our initial experiments (**Figure 1C, D**), we subsequently focused on investigating whether low levels of miR-6794-3p expression contribute to the invasion and migration of pancreatic cancer cells *in vitro* and *in vivo*. To confirm the above results, we analyzed the expression level of miR-6794-3p in HPAF-II, PANC-1, PATU-8988T, PATU-8898S, AsPC-1, and BxPC-3 cells. As shown in **Figure 1E**, miR-6794-3p was expressed at a high level in HPAF-II cells, but at low levels in the other pancreatic cancer cell lines. To evaluate the clinical significance of miR-6794-3p expression in pancreatic cancer, we determined the overall survival of pancreatic cancer patients with different levels of miR-6794-3p via Kaplan-Meier plot analysis (ENCORI for RNA interactomes database, http://rnasysu.com/encori/panMirSurvivalExp.php). Patients with low-level expression of miR-6794-3p had obviously shorter survival than patients with high-level miR-6794-3p expression (**Figure 1F**). These data indicate that low-level miR-6794-3p expression may be a novel biomarker for pancreatic cancer metastasis, and may also indicate the potential of miR-6794-3p as an inhibitor of pancreatic cancer metastasis.

### miR-6794-3p inhibits invasion, migration, and EMT in pancreatic cancer cells

**Figure 2:**
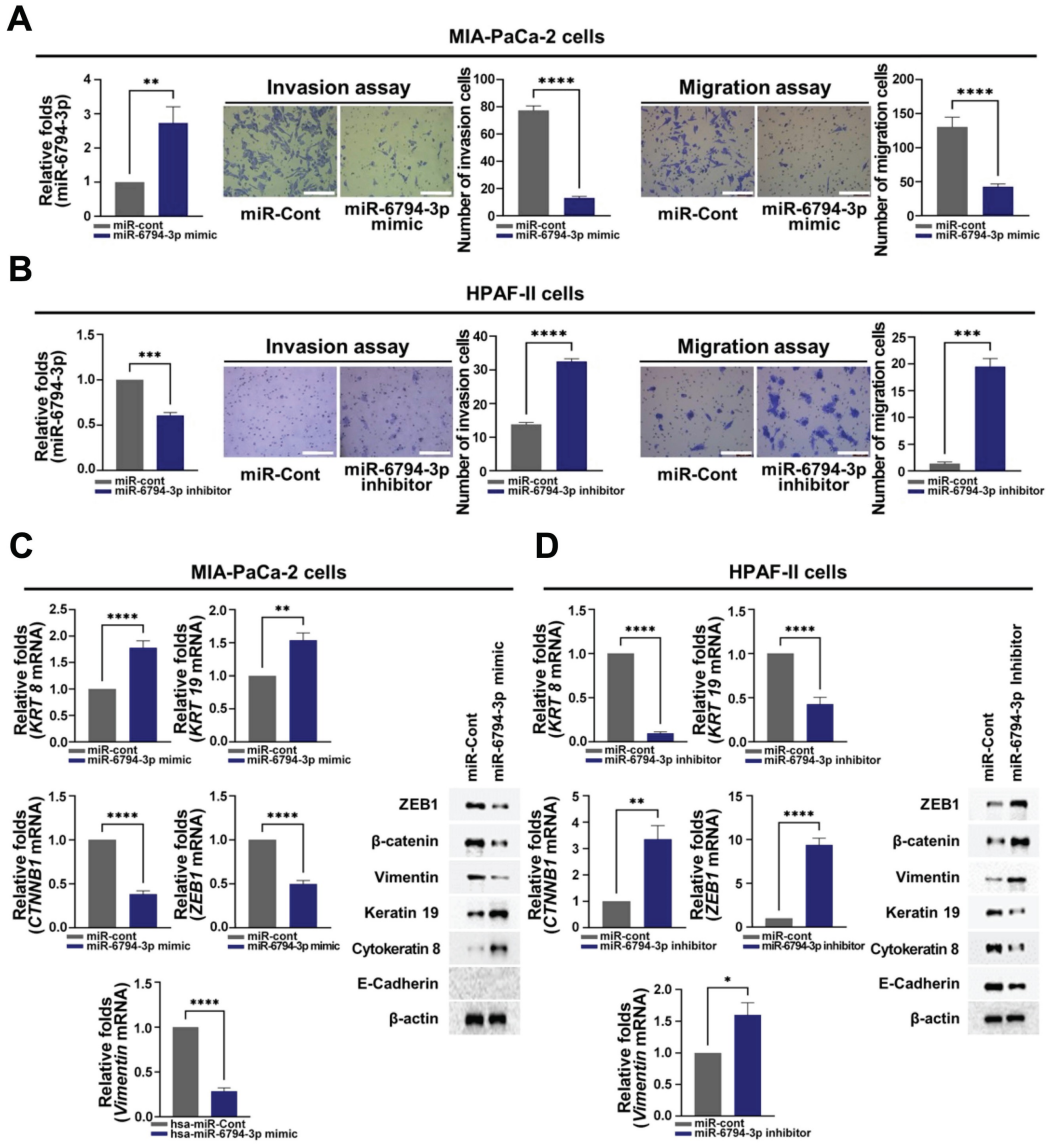
miR-6794-3p inhibits invasion, migration, and EMT of pancreatic cancer cells. **(A-B)** Invasion and migration assays on MIA-PaCa-2 **(A)** and HPFA-II **(B)** cells transfected with miR-Cont or miR-6794-3p mimic and miR-Cont or miR-6794-3p inhibitor, respectively. Left panel: Relative expression of miRNA-6794-3p in pancreatic cancer cells; Mid panel: Representative images and quantification results of invasion in pancreatic cancer cells; Right panel: Representative images and quantification results of migration in pancreatic cancer cells. All data are presented as mean ± SEM. * *P* < 0.001 with unpaired *t* test, ** *P* < 0.01 with unpaired *t* test, **** *P* < 0.0001 with unpaired *t* test. Bar = 200 μm **(C-D)** Expression of EMT (ZEB1, β-catenin and Vimentin) and MET markers (keratin 19 and cytokeratin 8) in MIA-PaCa-2 **(C)** and HPFA-II **(D)** cells transfected with miR-Cont or miR-6794-3p mimic and miR-Cont or miR-6794-3p inhibitor, respectively. Left panel: Relative mRNA expression of *KRT8*, *Vimentin*, *CTNNB1*, *Keratin 19*, and *ZEB1* in pancreatic cancer cells. Expression of mRNAs was evaluated via qPCR using *18S rRNA* as the internal control. All data are presented as mean ± SEM. * *P* < 0.05 with unpaired *t* test, ** *P* < 0.01 with unpaired *t* test, **** *P* < 0.0001 with unpaired *t* test. Right panel: Immunoblot analysis of the indicated protein levels.

The role of miR-6794-3p in pancreatic cancer metastasis has not been reported previously. Based on the results shown in **Figure 1E**, we performed gain- and loss-of-function studies using MIA-PaCa-2 and HPAF-II cells, which express low and high levels of miR-6794-3p, respectively. For the gain-of-function experiment, MIA-PaCa-2 cells were transfected with miR-6794-3p mimic or miR-Control (miR-Cont) and used for invasion and migration assays. Treatment with the miR-6794-3p mimic significantly inhibited invasion and migration relative to the control treatment (**Figure 2A**). Conversely, loss-of-function of miR-6794-3p in HPAF-II cells dramatically increased cell invasion and migration compared to the control treatment (**Figure 2B**). To validate these results, various pancreatic cancer cell lines with low-level endogenous expression of miR-6794-3p (i.e., PANC-1, PATU-8988T, PATU-8988S, AsPC-1, and BxPC-3 cells) were transfected with miR-6794-3p mimic or miR-Cont, and their invasion and migration properties were assessed. Similar to the data obtained with MIA-PaCa-2 cells, the miR-6794-3p mimic significantly inhibited the invasion and migration abilities of these cell lines compared to the control treatment (**Supplementary Figure S1**). Next, we investigated the effect of miR-6794-3p gain-of-function on the EMT signaling pathway in pancreatic cancer cells. Overexpression of miR-6794-3p in MIA-PaCa-2 cells enhanced the gene and protein expression levels of cytokeratin 8 and keratin 19 (epithelial markers) while suppressing those of β-catenin, vimentin and ZEB1 (mesenchymal markers) (**Figure 2C**). Conversely, depletion of miR-6794-3p in HPAF-II cells (i.e., loss of function) decreased the gene and protein expression levels of cytokeratin 8 and keratin 19 and increased those of vimentin, β-catenin, and ZEB1 (**Figure 2D**). E-cadherin is known as a typical epithelial marker of cells 35. However, it has been reported that E-cadherin is expressed at low levels in MIA-PaCa-2 cells 36. Consistent with that report, we found that E-cadherin is not expressed in MIA-PaCa-2 cells (**Supplementary Figure S2**).

Therefore, we did not analyze E-cadherin expression in MIA-PaCa-2 cells. A previous report has demonstrated that β-catenin, as a mesenchymal marker, induces EMT in cancer cells 37. Therefore, in this study, we observed whether β-catenin plays a role as a mesenchymal marker in pancreatic cancer cells. Indeed, we observed that siβ-catenin could inhibit pancreatic cancer cell migration and invasion (**Supplementary Figure S3**) and β-catenin could function as a mesenchymal marker in the pancreatic cancer cell lines. Together, our results clearly show that miR-6794-3p has inhibitory effects on the invasion, migration, and EMT of pancreatic cancer cells.

### miR-6794-3p targets *RBBP4* to suppress invasion, migration, and EMT in pancreatic cancer cells

**Figure 3:**
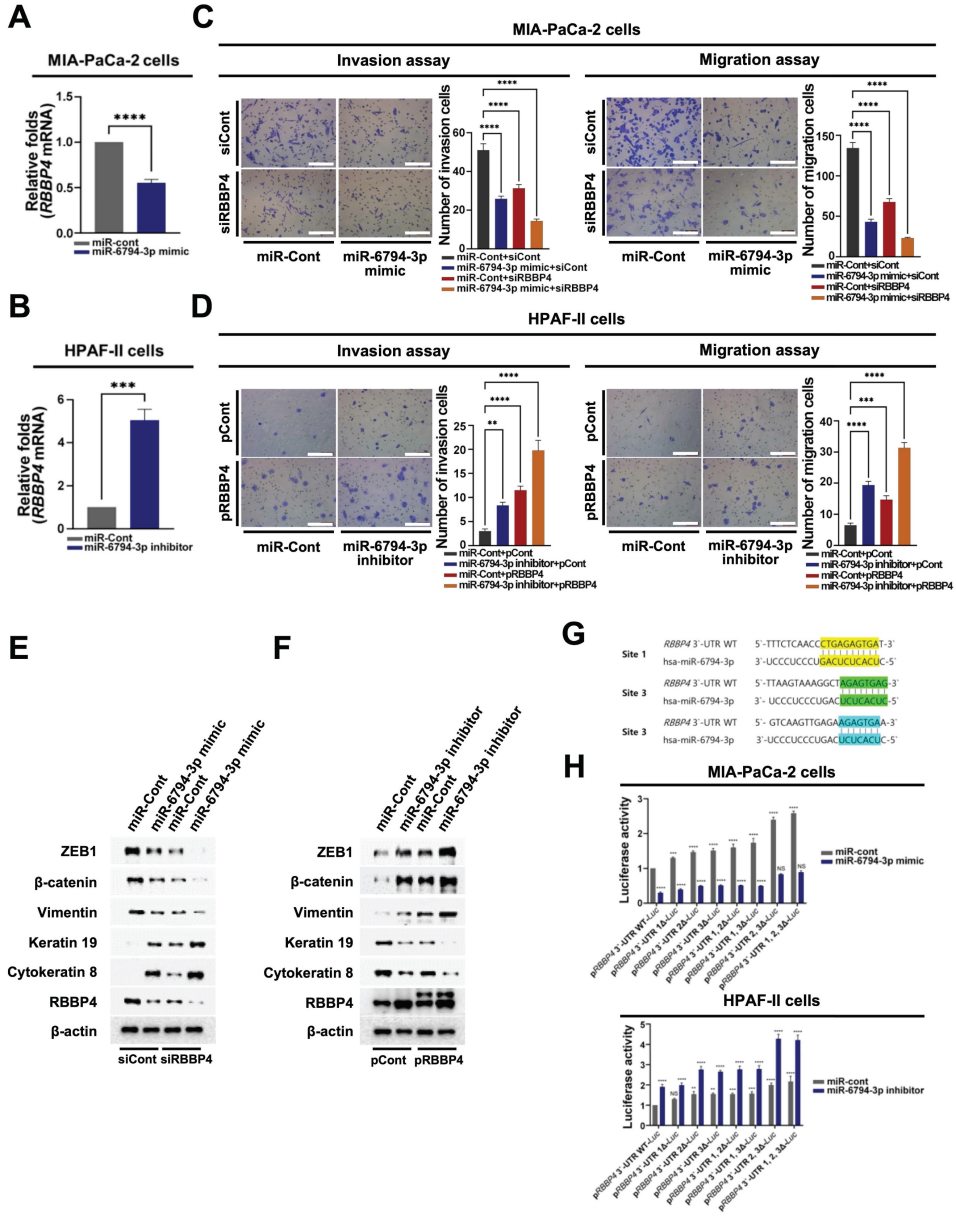
miR-6794-3p targets *RBBP4* to suppress invasion, migration, and EMT in pancreatic cancer cells.** (A-B)** Relative mRNA expression of *RBBP4* in MIA-PaCa-2 **(A)** and HPFA-II **(B)** cells transfected with miR-Cont or miR-6794-3p mimic and miR-Cont or miR-6794-3p inhibitor, respectively, examined via qPCR using *18S rRNA* as the internal control. All data are presented as mean ± SEM. *** *P* < 0.001 with unpaired *t* test, **** *P* < 0.0001 with unpaired *t* test. **(C)** Invasion and migration assays on MIA-PaCa-2 cells transfected with miR-Cont or miR-6794-3p mimic and siCont or siRBBP4. All data are presented as mean ± SEM. **** *P* < 0.0001 with unpaired *t* test. **(D)** Invasion and migration assays on HPAF-II cells transfected with miR-Cont or miR-6794-3p inhibitor and pCont or pRBBP4. All data are presented as mean ± SEM. ** *P* < 0.01 with unpaired *t* test, *** *P* < 0.001 with unpaired *t* test, **** *P* < 0.0001 with unpaired *t* test.** (E)** Expression of EMT and MET markers in MIA-PaCa-2 transfected with miR-Cont or miR-6794-3p mimic and siCont or siRBBP4. The indicated proteins were analyzed via immunoblot assay. **(F)** Expression of EMT and MET markers in MIA-PaCa-2 transfected with miR-Cont or miR-6794-3p mimic and siCont or siRBBP4. The indicated proteins were analyzed via immunoblot assay. **(G)** Alignment of miR-6794-3p with its corresponding complementary binding sequence in RBBP4 3'-UTR. **(H)** miR-Cont- or miR-6794-3p mimic-transfected MIA-PaCa-2 cells and miR-Cont- or miR-6794-3p inhibitor-transfected HPAF-II cells were transfected with control pRL-*Luc* and *RBBP4* 3′-UTR WT*-Luc* or *RBBP4* 3′-UTR 1Δ*-Luc* or *RBBP4* 3′-UTR 2Δ*-Luc* or *RBBP4* 3′-UTR 3Δ*-Luc* or *RBBP4* 3′-UTR 1, 2Δ*-Luc* or *RBBP4* 3′-UTR 1, 3Δ*-Luc* or *RBBP4* 3′-UTR 2, 3Δ*-Luc* or *RBBP4* 3′-UTR 1, 2, 3Δ*-Luc*. Luciferase activity was normalized to that of *Renilla*. All data are presented as mean ± SEM. ** *P* < 0.01 with ANOVA, *** *P* < 0.001 with ANOVA, **** *P* < 0.0001 with ANOVA. Ns indicates no significance.

To further establish the precise roles of miR-6794-3p in pancreatic cancer cells, we analyzed target genes using the TargetScan Human web server (https://www.targetscan.org/vert_80/) and miRBase database (https://www.mirbase.org/). This analysis identified *RBBP4*,* ATP6AP2*,* BCAT1, CA3*,* EPDR1*,* IL7*,* KIAA0087*,* NUDCD1*,* PAX4*,* PON2*,* TLNRD1*, and *TMCO1* as putative targets of miR-6794-3p. From among these genes, we focused on those reported to be involved in the modulation, invasion, migration, and/or EMT of pancreatic cancer cells. To confirm that the selected genes were functionally targeted by miR-6794-3p, MIA-PaCa-2 cells were transfected with miR-6794-3p mimic or miR-Cont and target gene expression was evaluated via qPCR. Transfection with the miR-6794-3p mimic inhibited the expression of *RBBP4*,* ATP6AP2, CA3*,* PON2*, and* TLNRD1* in MIA-PaCa-2 cells (**Figure 3A** and **Supplementary Figure S4A**). Conversely, miR-6794-3p inhibitor treatment of HPAF-II cells induced the expression of *RBBP4* and* BCAT1* (**Figure 3B** and **Supplementary Figure S4B**). To test the theory that miR-6794-3p acts as an upstream signal for *RBBP4* expression, we transfected siCont or siRBBP4 into MIA-PaCa-2 cells that had been transfected with miR-Cont or miR-6794-3p mimic, and transfected pCont or pRBBP4 into HPAF-II cells that had been transfected with miR-Cont or miR-6794-3p inhibitor, and then performed qPCR analysis of miR-6794-3p. As shown in **Supplementary Figure S4C and D**, siRBBP4 and pRBBP4 did not affect the miR-6794-3p level in MIA-PaCa-2 and HPAF-II cells, respectively. Based on these results, we inferred that* RBBP4* is a downstream target gene regulated by miR-6794-3p. Next, we investigated whether miR-6794-3p exerts its effects on invasion, migration, and EMT of pancreatic cancer cells through regulation of *RBBP4*. To this end, siCont or siRBBP4 was transfected into miR-6794-3p mimic or miR-Cont-transfected MIA-PaCa-2 cells, which were then analyzed for invasion, migration, and EMT signaling. Our results showed that the miR-6794-3p mimic or siRBBP4 inhibited the invasion and migration of pancreatic cancer cells, and their combined treatment exerted a greater inhibitory effect than either the miR-6794-3p mimic or siRBBP4 alone (**Figure 3C**). Invasion, migration and EMT were additionally examined in miR-6794-3p inhibitor- or miR-Cont-transfected HPAF-II cells treated with pCont or pRBBP4. As shown in **Figure 3D**, the miR-6794-3p inhibitor and pRBBP4 enhanced the invasion and migration of pancreatic cancer cells and their co-treatment exerted more pronounced effects than either agent alone (**Figure 3D**). Consistent with the data presented in **Figure 3C and D**, the expression levels of epithelial markers were decreased while those of mesenchymal markers were increased in pancreatic cancer cells showing enhanced invasion and migration (**Figure 3E, F**). To further explore the miR-6794-3p-mediated targeting and regulation of *RBBP4*, we sought to determine the sequences relevant for their binding. As shown in **Figure 3G**, miR-6794-3p interacted with three specific nucleotide sequences in the 3'-UTR of *RBBP4*. Accordingly, we focused on whether miR-6794-3p regulates *RBBP4* via binding its 3'-UTR region. To this end, we generated reporter plasmids containing the 3'-UTR of *RBBP4* (p*RBBP4* 3'-UTR WT-*Luc*) and deletion mutants of the predicted binding sites (*RBBP4* 3'-UTR 1Δ*-Luc*, *RBBP4* 3'-UTR 2Δ*-Luc*, *RBBP4* 3'-UTR 3Δ*-Luc*, *RBBP4* 3'-UTR 1, 2Δ*-Luc*, *RBBP4* 3'-UTR 1, 3Δ*-Luc*, *RBBP4* 3'-UTR 2, 3Δ*-Luc*, *RBBP4* 3'-UTR 1, 2, 3Δ*-Luc*). Each plasmid was transfected into MIA-PaCa-2 cells treated with or without miR-Cont or miR-6794-3p mimic and HPAF-II cells treated with or without miR-Cont or the miR-6794-3p inhibitor, and reporter activity was determined. Notably, luciferase activity was increased in the *RBBP4* 3'-UTR mutants with deletion of site 2 or 3 and highest in the RBBP4 3'-UTR mutant with deletion of both sites 2 and 3 (**Figure 3H**). Together, the above results indicate that miR-6794-3p regulates *RBBP4* expression through direct binding of its 3'-UTR region, and thereby modulates the invasion, migration, and EMT of pancreatic cancer cells.

### Expression of miR-6794-3p is silenced in pancreatic cancer cells via promoter hypermethylation

Next, we focused on the low-level expression of miR-6794-3p in pancreatic cancer cells showing enhanced invasion and migration, and the involvement of its suppressed expression in malignancy. To ascertain whether downregulation of miR-6794-3p in pancreatic cancer cells is due to promoter hypermethylation, we used methylation-specific polymerase chain reaction (MSP) to examine CpG island methylation in the miR-6794-3p promoter, and 5-aza-2'-deoxycytidine (5-Aza; an epigenetic regulator that causes DNA demethylation) to assess the impact of increased CpG island methylation in this promoter.

**Figure 4:**
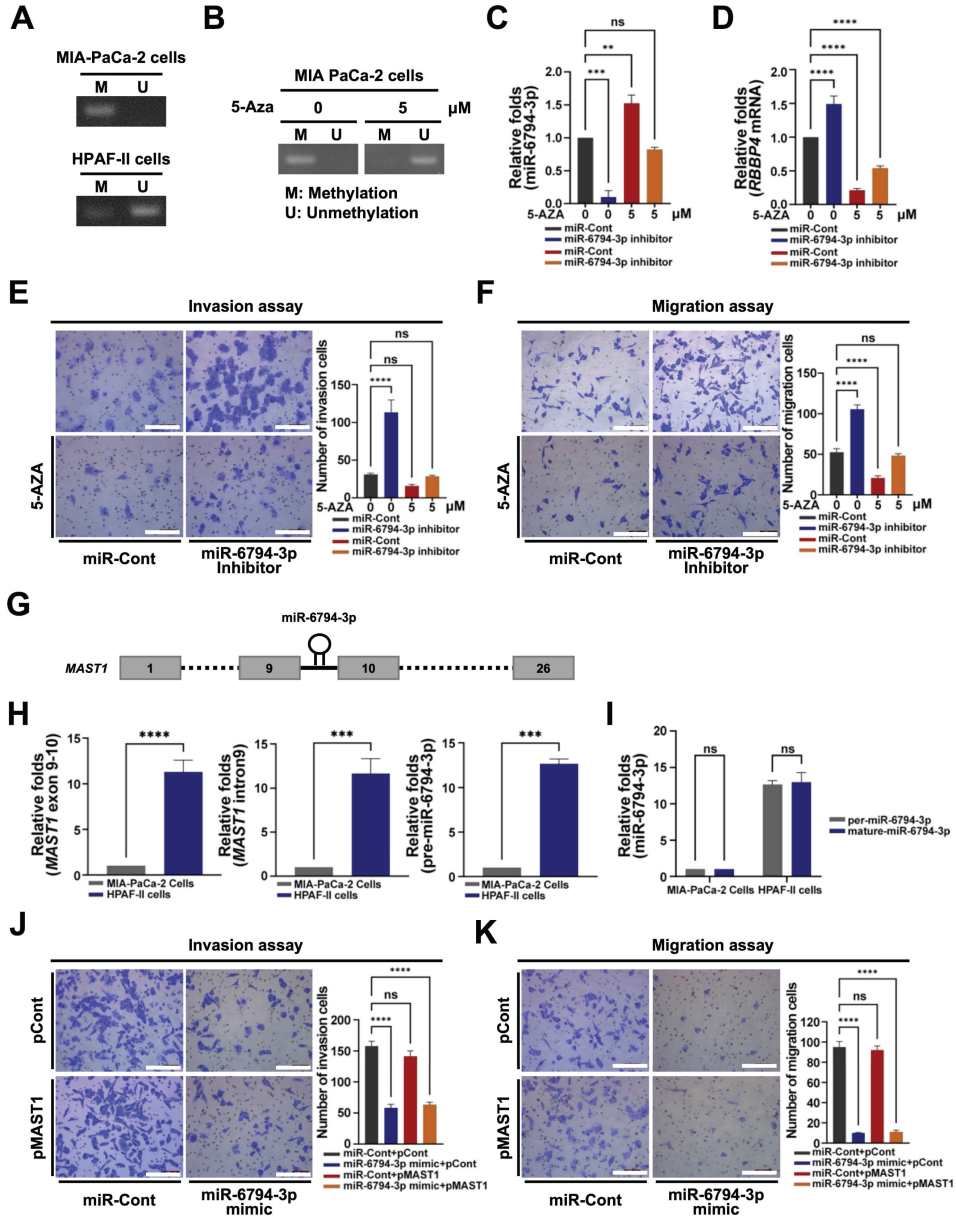
Expression of miR-6794-3p is silenced in pancreatic cancer cells via promoter hypermethylation.** (A)** Hypermethylation of the miR-6794-3p promoter in MIA-PaCa-2 and HPAF-II cells detected via methylation-specific PCR (MSP). **(B)** MSP of the miR-6794-3p promoter in MIA-PaCa-2 cells treated with 5-Aza for 5 days. **C** Relative levels of miR-6794-3p in miR-Cont- or miR-6793-3p inhibitor-transfected MIA-PaCa-2 cells treated with or without 5-Aza examined via qPCR using *U6 snRNA* as the internal control. All data are presented as mean ± SEM. ** *P* < 0.01 with unpaired *t* test, *** *P* < 0.001 with unpaired *t* test. Ns indicates no significance. **(D)** Relative mRNA levels of *RBBP4* in miR-Cont- or miR-6793-3p inhibitor-transfected MIA-PaCa-2 cells treated with or without 5-Aza examined via qPCR using *18S rRNA* as the internal control. All data are presented as mean ± SEM. **** *P* < 0.0001 with unpaired *t* test. **(E-F)** Invasion **(E)** and migration **(F)** assays of miR-Cont- or miR-6793-3p inhibitor-transfected MIA-PaCa-2 cells treated with or without 5-Aza. Left panel: Representative images of invading and migrating cells; Right panel: Quantification of invasion and migration. All data are presented as mean ± SEM. **** *P* < 0.0001 with unpaired *t* test. Ns indicates no significance. **(G)** Schematic presentation of miR-6794-3p in intron 9 of the MAST1 gene. **(H)** qPCR analysis of MAST1 exon 9-10, MAST1 intron 9, and pre-miR-6794 3p in MIA-PaCa-2 and HPAF-II cells using *U6 snRNA* as the internal control. All data are presented as mean ± SEM. *** *P* < 0.001 with unpaired *t* test, **** *P* < 0.0001 with unpaired *t* test. **(I)** qPCR expression of pre-miR-6794-3p and mature-miR-6794 3p in MIA-PaCa-2 cells and HPAF-II cells using *U6 snRNA* as the internal control. All data are presented as mean ± SEM. Ns indicates no significance. **(J-K)** Invasion **(J)** and migration **(K)** assays of miR-Cont- or miR-6793-3p mimic- and pCont- or pMAST1-transfected MIA-PaCa-2 cells. Left panel: Representative images of invasion and migration of cells; Right panel: Quantification of invasion and migration. All data are presented as mean ± SEM. **** *P* < 0.0001 with unpaired *t* test. Ns indicates no significance.

As shown in **Figure 4A**, methylation of CpG islands in the miR-6794-3p promoter was increased in MIA-PaCa-2 cells and decreased in HPFA-II cells. Furthermore, CpG island methylation in the miR-6794-3p promoter was reduced by 5-Aza treatment of MIA-PaCa-2 cells (**Figure 4B**). To investigate whether CpG island methylation in the miR-6794-3p promoter affects the expression of this miRNA and consequently that of its target gene, *RBBP4*, we transfected MIA-PaCa-2 cells with miR-Cont or miR-6794-3p inhibitor and treated the cells with or without 5-Aza. In miR-Cont-transfected MIA-PaCa-2 cells, which have a low endogenous level of miR-6794-3p, the expression of this miRNA was enhanced by 5-Aza and this change was suppressed by the miR-6794-3p inhibitor (**Figure 4C**). Consistently, 5-Aza treatment downregulated *RBBP4* in miR-Cont-transfected MIA-PaCa-2 cells while the miR-6794-3p inhibitor enhanced *RBBP4* expression (**Figure 4D**). To expand upon these findings, we examined the invasion and migration abilities of miR-Cont- or miR-6794-3p inhibitor-transfected MIA-PaCa-2 cells treated with or without 5-Aza. The miR-6794-3p inhibitor enhanced the invasion and migration of pancreatic cancer cells (**Figure 4E and F**). Treatment with 5-Aza inhibited the increase in invasion and migration of pancreatic cancer cells caused by the miR-6794-3p inhibitor (**Figure 4E and F**). To examine the details of miR-6794-3p expression in pancreatic cancer cells, we analyzed the genomic localization of the miR-6794-3p-encoding sequence, using the National Center for Biotechnology Information (NCBI) human genome database. We found that miR-6794-3p is encoded between exons 9 and 10 of the microtubule-associated serine/threonine kinase 1 (*MAST1*) gene located on chromosome 19 (**Figure 4G**). Accordingly, we further investigated the effects of *MAST1*, the host gene of miR-6794-3p, on pancreatic cancer cell invasion and migration. First, we compared the expression patterns of *MAST1* exon 9-10, *MAST1* intron 9, and pre-miR-6794-3p between MIA-PaCa-2 and HPAF-II cell groups. All three gene regions showed low-level expression in MIA-PaCa-2 cells and high-level expression in HPAF-II cells (**Figure 4H**). To further establish the specific location of miR-6794-3p between exons 9 and 10 of the *MAST1* gene on chromosome 19, the expression levels of pre-miR-6794-3p and mature miR-6794-3p were evaluated in MIA-PaCa-2 and HPAF-II cells. The levels of both pre-miR-6794-3p and mature miR-6794-3p were low in MIA-PaCa-2 and high in HPAF-II cells (**Figure 4I**). The levels of pre-miR-6794-3p and mature miR-6794-3p were similar in HPAF-II cells (**Figure 4I**), further suggesting that miR-6794-3p is specifically located and expressed in *MAST1* of chromosome 19. Since miR-6794-3p is located between exons 9 and 10 of *MAST1*, we investigated whether expression of the host gene could affect pancreatic cancer cell invasion and migration. To this end, *MAST1* was overexpressed in MIA-PaCa-2 cells transfected with miR-Cont or miR-6794-3p mimic, and the invasion and migration properties of the cells were assessed. We found that overexpression of *MAST1* in pancreatic cancer cells did not affect the gene expression of miR-6794-3p, or vice versa (**Supplementary Figure S5**). Moreover, *MAST1* upregulation did not restore the miR-6794-3p-induced suppression of pancreatic cancer cell invasion and migration (**Figure 4J, K**). Based on these collective results, we conclude that miR-6794-3p is positioned between exons 9 and 10 of *MAST1* located on chromosome 19, and its expression is regulated via methylation of CpG islands in the host gene promoter. Furthermore, expression of *MAST1* does not alter the invasion or migration of pancreatic cancer cells.

### miR-6794-3p inhibits methylation of histone H3 through inhibition of *RBBP4* in pancreatic cancer cells

**Figure 5:**
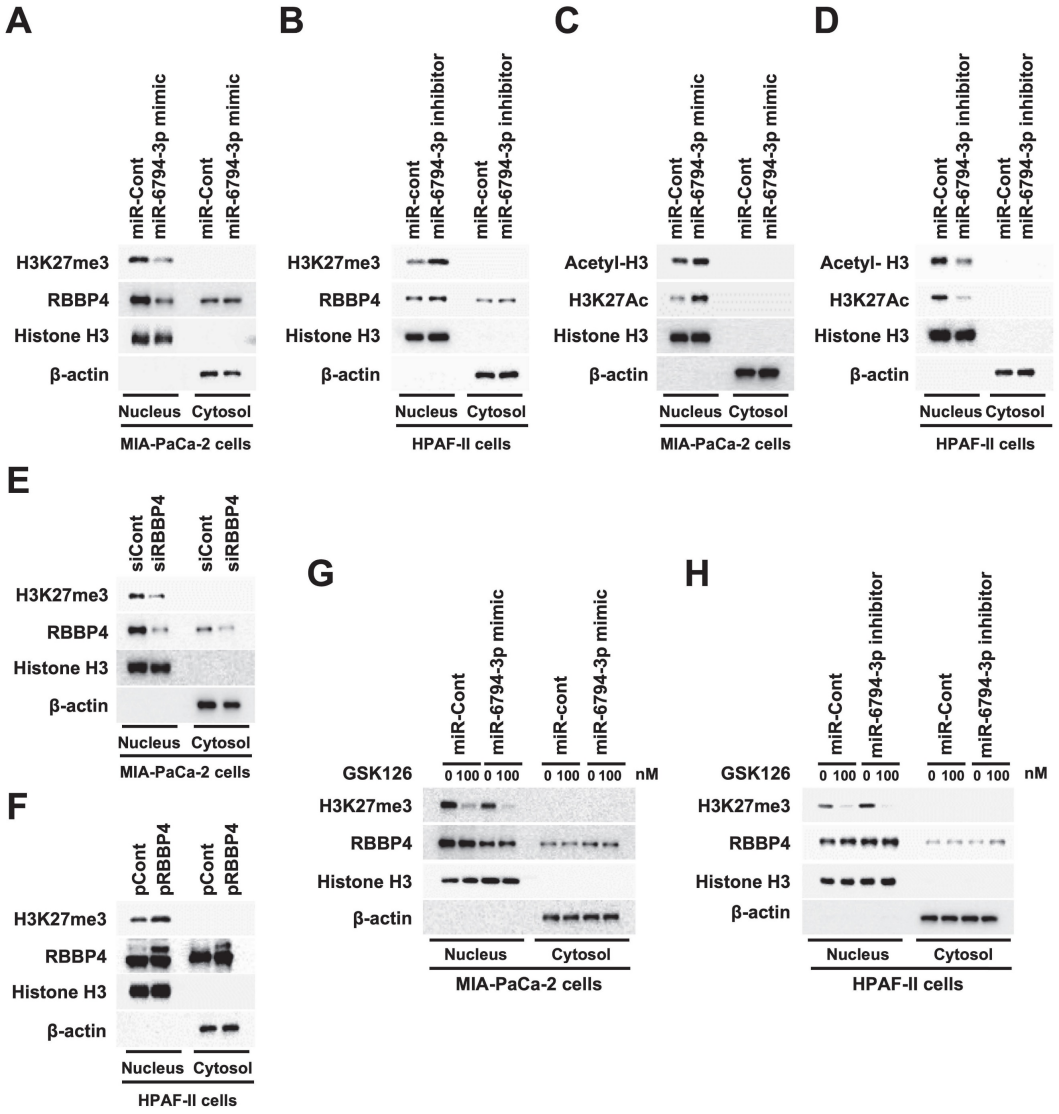
miR-6794-3p inhibits methylation of histone H3 through inhibiton of *RBBP4* expression in pancreatic cancer cells.** (A-B)** Methylation of histone H3 in miR-Cont- or miR-6794-3p mimic-transfected MIA-PaCa-2 cells **(A)** and miR-Cont- or miR-6794-3p inhibitor-transfected HPAF-II cells **(B)**. The indicated protein levels were analyzed via immunoblot assay. **(C-D)** Acetylation of histone H3 in miR-Cont- or miR-6794-3p mimic-transfected MIA-PaCa-2 cells **(C)** and miR-Cont- or miR-6794-3p inhibitor-transfected HPAF-II cells **(D)**. The indicated protein levels were analyzed via immunoblot assay.** (E-F)** Methylation of histone H3 in siCont- or siRBBP4- transfected MIA-PaCa-2 cells **(E)** and pCont- or pRBBP4- transfected HPAF-II cells **(F)**. Immunoblot analysis of the indicated protein levels. **(G-H)** Methylation of histone H3 in miR-Cont- or miR-6794-3p mimic-transfected MIA-PaCa-2 cells **(G)** treated with or without GSK126 and miR-Cont- or miR-6794-3p inhibitor-transfected HPAF-II cells treated with or without GSK126 **(H)**. The indicated protein levels were analyzed via immunoblot assay.

RBBP4, a component of polycomb repressive complex 2 (PRC2), plays an important role in methylation of histone H3 via binding of the PRC2 complex to chromatin, thereby leading to chromatin condensation and inhibition of gene expression 19,20. Here, we examined the hypothesis that miR-6794-3p-mediated inhibition of RBBP4 enhances histone H3 acetylation, leading to increased expression of epithelial markers and, consequently, inhibition of pancreatic cancer cell invasion and migration. Methylation of histone H3 in miR-Cont- or miR-6794-3p mimic-transfected MIA-PaCa-2 and miR-Cont- or miR-6794-3p inhibitor-transfected HPAF-II cells was investigated. As shown in **Figure 5A**, the miR-6794-3p mimic inhibited *RBBP4* expression and methylation of histone H3 in MIA-PaCa-2 cells. Conversely, the miR-6794-3p inhibitor increased *RBBP4* expression and histone H3 methylation in HPAF-II cells (**Figure 5B**).

In keeping with these results, the miR-6794-3p mimic increased acetylation of histone H3 in MIA-PaCa-2 cells (**Figure 5C**) while the inhibitor decreased acetylation of histone H3 in HPAF-II cells (**Figure 5D**). To further confirm these findings, methylation of histone H3 was evaluated in siCont- or siRBBP4- transfected MIA-PaCa-2 and pCont- or pRBBP4-transfected HPAF-II cells. Notably, siRBBP4 suppressed methylation of histone H3 in MIA-PaCa-2 cells (**Figure 5E**) and, conversely, pRBBP4 increased methylation of histone H3 in HPAF-II cells (**Figure 5F**). Next, we investigated whether miR-6794-3p affects the major complex of PRC2 and regulates histone H3 methylation, regardless of *RBBP4* expression. Enhancer of zeste homologue 1/2 (EZH1/2), a major subunit of PRC2, suppresses transcription via lysine 27 methylation of histone H3 20. Accordingly, we assessed methylation of histone H3 and expression of *RBBP4* in miR-Cont- or miR-6794-3p mimic-transfected MIA PaCa-2 cells and miR-Cont- or miR-6794-3p inhibitor-transfected HPAF-II cells treated with the EZH2 inhibitor GSK126. Both methylation of histone H3 and *RBBP4* expression in pancreatic cancer cells were significantly inhibited by miR-6794-3p while GSK126 only inhibited histone H3 methylation (**Figure 5G-H**). The overall results suggest that miR-6794-3p inhibits methylation of histone H3 by suppressing *RBBP4* expression, thereby uncovering a potential mechanism underlying the involvement of histone H3 methylation in invasion and migration of pancreatic cancer cells.

### miR-6794-3p-mediated inhibition of RBBP4 regulates GRHL2 expression and pancreatic cancer cell invasion, migration and EMT

**Figure 6:**
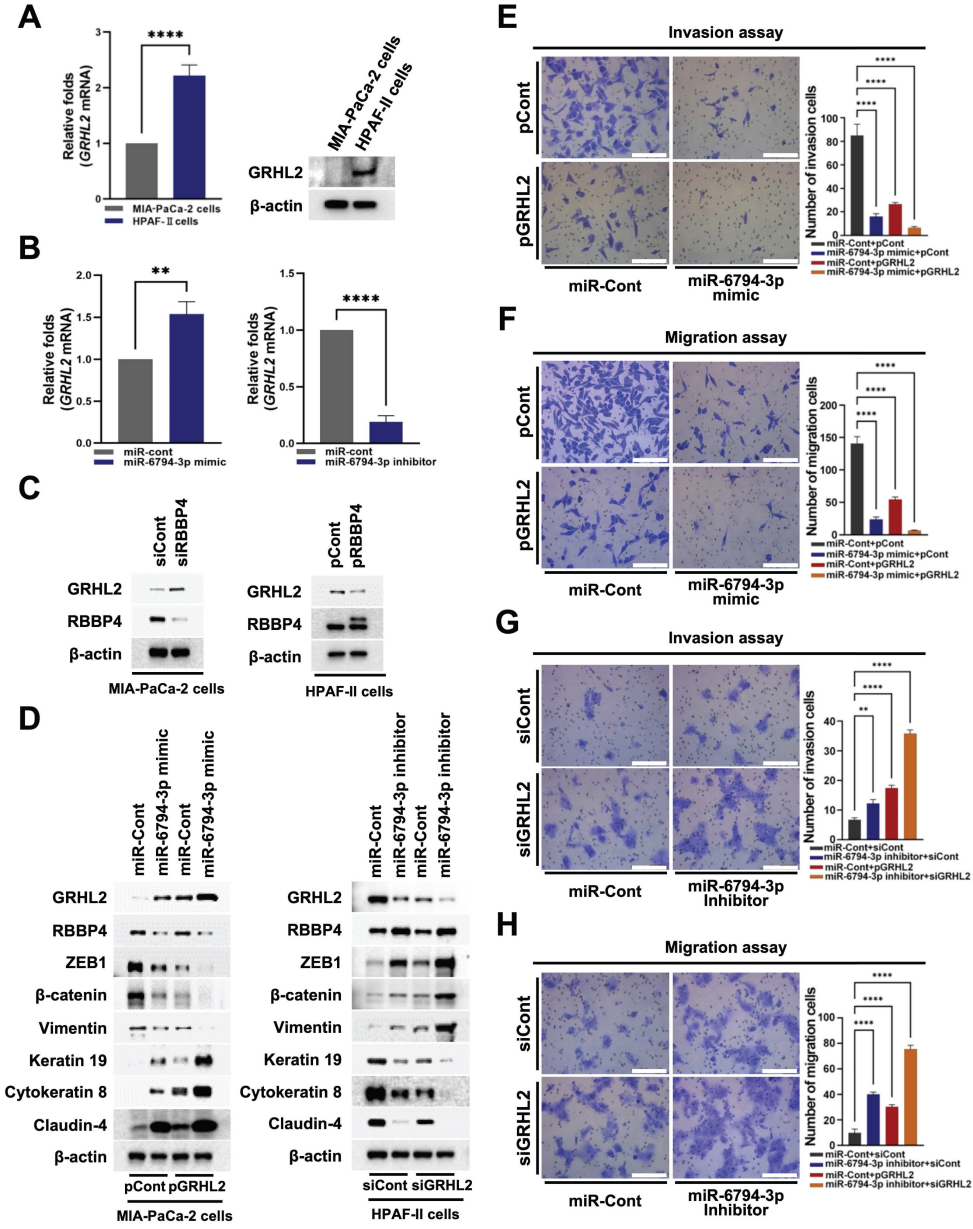
miR-6794-3p-mediated inhibition of *RBBP4* regulates *GRHL2* expression and pancreatic cancer cell invasion and migration. **(A)** Expression of *GRHL2* in MIA-PaCa-2 and HPFA-II cells. Left panel: Relative mRNA expression of *GRHL2* in MIA-PaCa-2 and HPFA-II cells examined via qPCR using 18S rRNA as the internal control. All data are presented as mean ± SEM. **** *P* < 0.0001 with unpaired t test. Right panel: Immunoblot analysis of the indicated protein levels. **(B)** Relative mRNA expression of *GRHL2* in miR-Cont- or miR-6794-3p mimic-transfected MIA-PaCa-2 cells (left panel) and miR-Cont- or miR-6794-3p inhibitor-transfected HPFA-II cells (right panel) examined via qPCR using 18S rRNA as the internal control. All data are presented as mean ± SEM. ** P < 0.01 with unpaired t test, **** *P* < 0.0001 with unpaired t test. **(C)** Expression of RBBP4 and GRHL2 in siCont- or siRBBP4-transfected MIA-PaCa-2 cells (left panel) and pCont- or pRBBP4-transfected HPAF-II cells (right panel).** (D)** Expression of EMT and MET markers in miR-Cont- or miR-6794-3p mimic- and pCont- or pGRHL2-transfected MIA-PaCa-2 cells (left panel) and miR-Cont- or miR-6794-3p inhibitor- and siCont- or siGRHL2-transfected HPAF-II cells (right panel).** (E-F)** Invasion **(E)** and migration **(F)** assays of miR-Cont- or miR-6793-3p mimic- and pCont- or pGRHL2-transfected MIA-PaCa-2 cells. Left panel: Representative images of invasion and migration of cells. Right panel: Quantification of invasion and migration. All data are presented as mean ± SEM. **** *P* < 0.0001 with unpaired t test. **(G-H)** Invasion **(G)** and migration **(H)** assays of miR-Cont- or miR-6793-3p inhibitor- and siCont- or siGRHL2-transfected HPAF-II cells. Left panel: Representative images of invasion and migration of cells. Right panel: Quantification of invasion and migration. All data are presented as mean ± SEM. ** *P* < 0.01 with unpaired t test, **** *P* < 0.0001 with unpaired t test.

Cancer cells display EMT/MET plasticity in the form of intermediate/hybrid/metastable states with co-existing epithelial and mesenchymal features 31. The changes in EMT/MET suggest that regulatory circuits among transcription factors (TFs) involve complex interplay between EMT inducers (SNAI1/2, ZEB1/2, TWIST1) and EMT suppressors (GRHL2, OVOL1/2) 38. Some TFs are major players in epigenetic remodeling, such as DNA methylation and histone modification 38. GRHL2 is known to regulate epigenetic remodeling and EMT status 38. In addition, GRHL2 acts as a dual transcription factor that induces apical junction proteins, such as E-cadherin and claudin-4, while suppressing EMT-related genes, such as *ZEB1* 38. Accordingly, we examined whether miR-6794-3p regulates GRHL2-mediated pancreatic cancer cell invasion and migration through regulatory effects on RBBP4. Expression of GRHL2 was initially determined in MIA-PaCa-2 and HPAF-II cell lines. GRHL2 mRNA and protein levels were low in MIA-PaCa-2 cells and high in HPFA-II (**Figure 6A**). Subsequently, we investigated the effects of miR-6794-3p on GRHL2 expression in pancreatic cancer cells. For these experiments, MIA-PaCa-2 cells were transfected with miR-Cont or miR-6794-3p mimic and HPAF-II cells with miR-Cont or miR-6794-3p inhibitor. The miR-6794-3p mimic increased *GRHL2* levels in MIA-PaCa-2 cells, and conversely, the inhibitor suppressed *GRHL2* in HPAF-II cells (**Figure 6B**). To explore the signal cascade between miR-6794-3p and GRHL2 in pancreatic cancer cells, we transfected miR-Cont- or miR-6794-3p mimic-MIA-PaCa-2 cells and miR-Cont- or miR-6794-3p inhibitor-transfected HPAF-II cells with pCont or pGRHL2 and siCont or siGRHL2, respectively. Expression of GRHL2 exerted no effect on miR-6794-3p expression (**Supplementary Figure S6A-B**). To uncover the regulatory associations between RBBP4 and GRHL2 in pancreatic cancer cells, MIA-PaCa-2 and HPAF-II cells were transfected with siCont or siRBBP4 and pCont or pRBBP4, respectively. Notably, inhibition of RBBP4 increased GRHL2 expression in MIA-PaCa-2 cells while its overexpression suppressed GRHL2 expression in HPAF-II (**Figure 6C**). Next, we determined whether miR-6794-3p-mediated inhibition of *RBBP4* expression exerts a regulatory effect on *GRHL2* in pancreatic cancer cells. Overexpression of *RBBP4* restored miR-6794-3p-mediated inhibition of *GRHL2* in MIA-PaCa-2 cells (**Supplementary Figure S7A**). In contrast, inhibition of *RBBP4* reduced miR-6794-3p-mediated increase of GRHL2 expression in HPAF-II (**Supplementary Figure S7B**). We further investigated whether miR-6794-3p-mediated inhibition of *RBBP4* expression enhances the *GRHL2*-induced increase in expression of epithelial markers and decrease in invasion and migration in pancreatic cancer cells. Overexpression of *GRHL2* restored miR-6794-3p mimic-mediated inhibition of epithelial marker expression and reduced mesenchymal marker expression in MIA-PaCa-2 cells (**Figure 6D**, left panel). Conversely, treatment with siGRHL2 reduced miR-6794-3p inhibitor-mediated increase in epithelial marker expression and restored mesenchymal marker expression in HPAF-II cells (**Figure 6D**, right panel). Consistent with these results, we confirmed that miR-6794-3p-mediated inhibition of *RBBP4* increases *GRHL2* expression, in turn, suppressing pancreatic cancer cell invasion and migration (Figure 6E-H). RBBP4 is a component of several chromatin-modifying structures, including the PRC2 complex 19. The PRC2 complex is recruited to CpG islands of unmethylated target gene promoters 39. The recruited PRC2 complex begins to perform the methylation process on the target gene 39. Therefore, we investigated RBBP4 binding to promoter on the *GRHL2* using chromatin immunoprecipitation (ChIP) assays.

To this end, DNA from MIA-PaCa-2 cells incubated for 16 h was crosslinked, extracted, and incubated with anti-RBBP4 antibody or control antibody (anti-IgG). RBBP4/DNA complexes were immunoprecipitated and crosslinking was reversed, followed by PCR amplification targeting promoter of GRHL2. Complexes immunoprecipitated with the anti-RBBP4 antibody generated a PCR band, confirming association with GRHL2 promoter, whereas those obtained with the control antibody produced no PCR bands (**Supplementary Figure S8**).

### miR-6794-3p inhibits metastatic activity of pancreatic cancer cells *in vivo*

**Figure 7:**
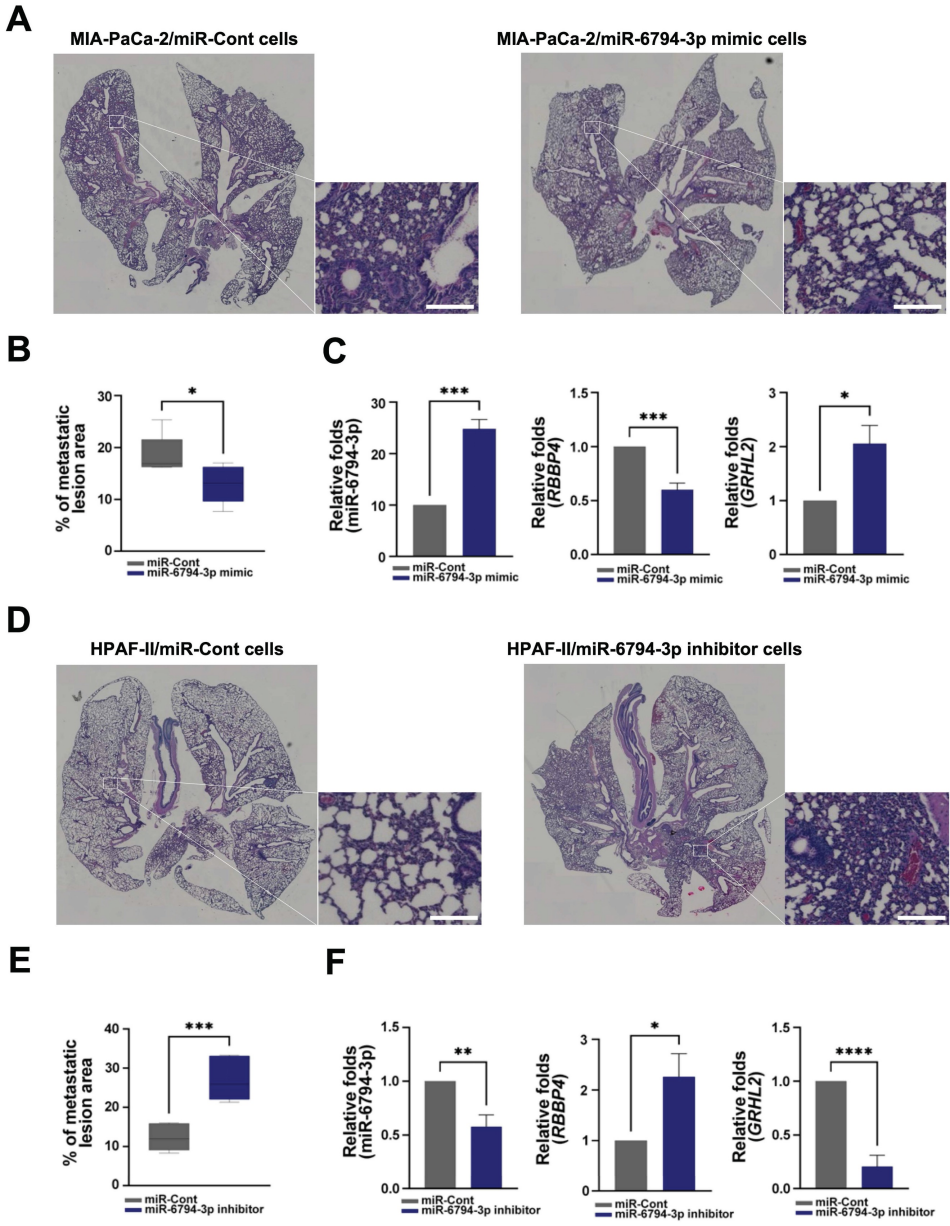
miR-6794-3p inhibits metastatic activity of pancreatic cancer cells *in vivo*. **(A)** Representative images of lung metastasis and H&E-stained lung sections in miR-Cont- or miR-6794-3p mimic- transfected MIA-PaCa-2 cells. Bar = 200 μm. **(B)** Metastatic lesion area of miR-Cont- or miR-6794-3p mimic- transfected MIA-PaCa-2 cells. All data are presented as mean ± SEM. * P < 0.05 with unpaired t test **(C)** Relative RNA expression of *RBBP4* and *GRHL2* in lung metastatic tissues of miR-Cont- or miR-6794-3p mimic- transfected MIA-PaCa-2 cells. All data are presented as mean ± SEM. * *P* < 0.05 with unpaired t test, *** *P* < 0.001 with unpaired t test. **(D)** Representative images of lung metastasis and H&E-stained lung sections in miR-Cont- or miR-6794-3p inhibitor-transfected HPFA-II cells. Bar = 200 μm. **(E)** Metastatic lesion area of miR-Cont- or miR-6794-3p inhibitor-transfected HPFA-II cells. All data are presented as mean ± SEM. *** *P* < 0.001 with unpaired t test **(F)** Relative RNA expression of *RBBP4* and *GRHL2* in lung metastatic tissues of miR-Cont- or miR-6794-3p inhibitor-transfected HPFA-II cells. All data are presented as mean ± SEM. * *P* < 0.05 with unpaired t test, ** *P* < 0.01 with unpaired t test, **** *P* < 0.0001 with unpaired t test.

Finally, for *in vivo* evaluation of the anti-metastatic activity of miR-6794-3p, we injected pancreatic cancer cells with stable knockdown or overexpression of miR-6794-3p into the tail veins of nude mice. In the lung metastasis model, the metastatic region injected with MIA-PaCa-2/miR-6794-3p mimic cells was smaller than that in mice injected with MIA-PaCa-2/miR-Cont cells (**Figure 7A-B**). miR-6794-3p mimic effectively inhibited metastatic region in the lung metastasis (**Figure 7A-B**). Consistent with these results, we used qPCR to confirm that miR-6794-3p mimic-mediated inhibition of *RBBP4* increased *GRHL2* expression in lung tissue when injected with MIA-PaCa-2/miR-6794-3p mimic cells in the metastasis model (**Figure 7C**). In contrast, the metastatic region in mice injected with HPAF-II/miR-6794-3p inhibitor cells was larger relative to that in mice injected with HPAF-II/miR-Cont cells (**Figure 7D-E**). miR-6794-3p inhibitor increased metastatic region in the lung metastasis (**Figure 7D-E**). Consistent with these results, we used qPCR to confirm that miR-6794-3p inhibitor-mediated increase of *RBBP4* inhibited *GRHL2* expression in lung tissue when injected with HPAF-II/miR-6794-3p inhibitor cells in the metastasis model (**Figure 7F**). These *in vivo* results support an important role of miR-6794-3p in pancreatic metastasis.

## Discussion

Here, we identified a mechanism by which miR-6794-3p suppresses pancreatic cancer metastasis. Mechanistic analyses revealed that miR-6794-3p inhibits RBBP4 by directly binding to the 3′-UTR of *RBBP4* and thereby upregulating GRGL2 to suppress pancreatic cancer cell invasion, migration, and metastasis. Expression of miR-6794-3p is suppressed by hypermethylation in the CpG island promoter region.

Pancreatic cancer remains a major clinical concern because it resists most available treatment options and there is no effective means for early diagnosis 9. Serum carbohydrate antigen 19-9 (CA 19-9) is commonly used as an indicator for clinical treatment efficacy 11; it suffers from ineffectiveness, low sensitivity, and low specificity, but is currently the only FDA-approved marker for pancreatic cancer 11. It is hoped that elucidating the molecular mechanisms underlying disease progression could facilitate the development of more efficient diagnostic and therapeutic strategies. Increasing interest has focused on exploring the utility of miRNAs for the early diagnosis and/or treatment of pancreatic cancer. miRNAs may function as tumor suppressors or oncogenes, and have been linked to various cancer types 9. Multiple miRNAs, including miRNA-483-3p, miRNA-21 and miRNA-155, have been identified as biomarkers of pancreatic ductal adenocarcinoma 12,14. A few studies have linked certain miRNAs, including miR-301a, miRNA-148a, miR-186, and miRNA-32, with the metastasis of pancreatic cancer 15-17. However, although these earlier reports found differences in the expression and function of miRNAs in normal versus pancreatic cancer tissues, no published study offers evidence of their direct effects on metastasis. Therefore, to identify whether miRNAs are directly involved in the metastasis of pancreatic cancer tissues, we herein first focused on analyzing miRNA expression patterns in metastatic and non-metastatic pancreatic cancer tissues.

Microarray analysis revealed 1.5-fold increases in the expression levels of miR-181-5p, miR-4454, miR-99b-3p, miR-1261, miR-3065-5p, miR-7975, and miR-1913, and 1.5-fold decreases in miR-3652, miR-4449, miR-190a-3p, and miR-6794-3p in metastatic versus non-metastatic pancreatic tissues samples (**Figure 1A**). We observed no changes in the expression levels of miRNA-483-3p, miRNA-21, miRNA-155, miR-301a, miRNA-148a, miR-186, and miRNA-326 12-17 between the metastatic and non-metastatic groups (**Figure 1A**). Using qPCR analysis of tissue samples and invasion and migration assays of pancreatic cancer cell lines treated with mimics or inhibitors of selected miRNAs, we found that low levels of miR-6794-3p expression were associated with pancreatic cancer cell invasion and migration (**Figure 1B-D**).

To further explore this association, we performed gain-of-function analyses in MIA-PaCa-2, PANC-1, PATU-8988T, PATU-8898S, AsPC-1, and BxPC-3 cells with low endogenous miR-6794-3p expression and loss-of-function analyses in HPAF-II cells with high endogenous miR-6794-3p expression. Notably, transfection with the miR-6794-3p mimic significantly inhibited the invasion, migration, and EMT signaling of pancreatic cancer cells expressing low levels of miR-6794-3p (**Supplementary Figure S1, Figure 2A**), whereas the miR-6794-3p inhibitor increased the invasion, migration, EMT signaling, and mesenchymal marker expression of HPAF-II cells (**Figure 2B, D**). These results suggested that miR-6794-3p could act as a tumor suppressor in pancreatic cancer cells by negatively affecting aspects of their metastatic ability, including invasion, migration, and EMT signaling.

Accordingly, we set out to comprehensively investigate the pathways through which miR-6794-3p inhibits the invasion, migration, and EMT signaling of pancreatic cancer cells. Among the putative miR-6794-3p target genes identified by our bioinformatics analysis, we selected 12 that are believed to be related to cancer metastasis. Experiments performed using MIA-PaCa-2 and HPAF-II cells transfected with a miR-6794-3p mimic and inhibitor, respectively, confirmed *RBBP4* as a target of miR-6794-3p (**Supplementary Figure S4**). The encoded RBBP4 is a putative functional component of the retinoblastoma protein complex (Rb) 40 and a member of several histone deacetylase (HDAC) complexes 41 involved in chromatin remodeling, including chromatin assembly factor 1 (CAF-1) 42, Sin3 complex 42, and nucleosome remodeling and deacetylation (NuRD) complex 43. The role of RBBP4 in tumor development has been a topic of significant research interest in recent decades 44. Aberrant expression of RBBP4 has been implicated in the poor prognosis and metastasis of several highly invasive tumor types, such as colon cancer 21, neuroblastoma 22, and lung cancer 23. However, its contribution to metastasis in pancreatic cancer was unknown. The miRNA-mediated regulation of RBBP4 has been associated with cell proliferation of non-small cell lung cancer 25 and carcinogenesis of glioblastoma 26. In pancreatic cancer, in contrast, no previous study had identified an *RBBP4*-targeting miRNA with implications in cancer cell invasion and migration. Based on the above-described experiments, we investigated whether miR-6794-3p could be involved in regulating the invasion, migration, and EMT of pancreatic cancer cells via changes in *RBBP4*. Indeed, we found that the miR-6794-3p mimic inhibited the invasion, migration, and EMT signaling of MIA-PaCa-2 cells by suppressing RBBP4 expression (**Figure 3A, C, E**), whereas the miR-6794-3p inhibitor increased these metastasis-related parameters in HPAF-II cells via upregulation of RBBP4 (**Figure 3B, D, F**). Based on the observation that miRNAs can inhibit the expression of target genes by binding the 3'-UTRs of mRNAs 9, we performed luciferase reporter assays and identified three miR-6794-3p-binding sites within the 3'-UTR of the *RBBP4* mRNA (**Figure 3G, H**). Our collective findings support the notion that miR-6794-3p acts as a tumor suppressor in pancreatic cancer cells through targeting *RBBP4*.

Changes in EMT/MET are related to the balance among TFs, EMT inducers, and EMT repressors 38. The TF, GRHL2, is known to regulate epigenetic remodeling and EMT status 38. As mentioned above, RBBP4 is involved in nucleosome remodeling. Our experiments designed to determine whether miR-6794-3p-regulated RBBP4 participates in nucleosome remodeling revealed that the miR-6794-3p-mediated suppression of RBBP4 effectively inhibited H3K27 acetylation in pancreatic cancer cells** (Figure 5A-F)**. We also investigated whether miR-6794-3p-inhibited RBBP4 signaling could regulate the invasion and migration of pancreatic cancer cells by modulating GRHL2 expression. Indeed, we found that: GRHL2 expression was low in MIA-PaCa-2 cells and high in HPAF-II cells (**Figure 6A**); miR-6794-3p mimic-mediated downregulation of RBBP4 increased GRHL2 expression and decreased invasion, migration, and EMT signaling in MIA-PaCa-2 cells (**Figure 6B-F**); and miR-6794-3p inhibitor-mediated upregulation of RBBP4 decreased GRHL2 expression and promoted invasion, migration and EMT signaling in HPAF-II cells (**Figure 6B-D, G, H**). Together, these results suggest that the tumor suppressor activity of miR-6794-3p is exerted via the ability of RBBP4 inhibition to upregulate GRHL2.

Low expression of tumor suppressor miRNAs in a number of human tumor types is primarily attributed to hypermethylation in the CpG island promoter of the primary transcript, which facilitates cancer growth 45,46. Using the NCBI human genome database, we localized the sequence encoding miR-6794-3p to a region between exons 9 and 10 of the microtubule-associated serine/threonine kinase 1 (*MAST1*) gene on chromosome 19 (**Figure 4G**). Accordingly, we examined the effects of *MAST1* on pancreatic cancer cell invasion and migration. However, we found that *MAST1* and its encoded protein product did not appear to be associated with the invasion or migration of pancreatic cancer cells (**Figure 4J, K**). Experiments using 5-Aza to inhibit DNA methylation showed that miR-6794-3p expression is regulated via promoter methylation of *MAST1* in MIA-PaCa-2 cells (**Figure 4B, C**), and that 5-Aza treatment inhibited *RBBP4* expression and invasion/migration in MIA-PaCa-2 cells (**Figure 4D-F**). These data collectively suggest that miR-6794-3p negatively regulates metastasis, and thereby acts as a tumor suppressor.

**Figure 8:**
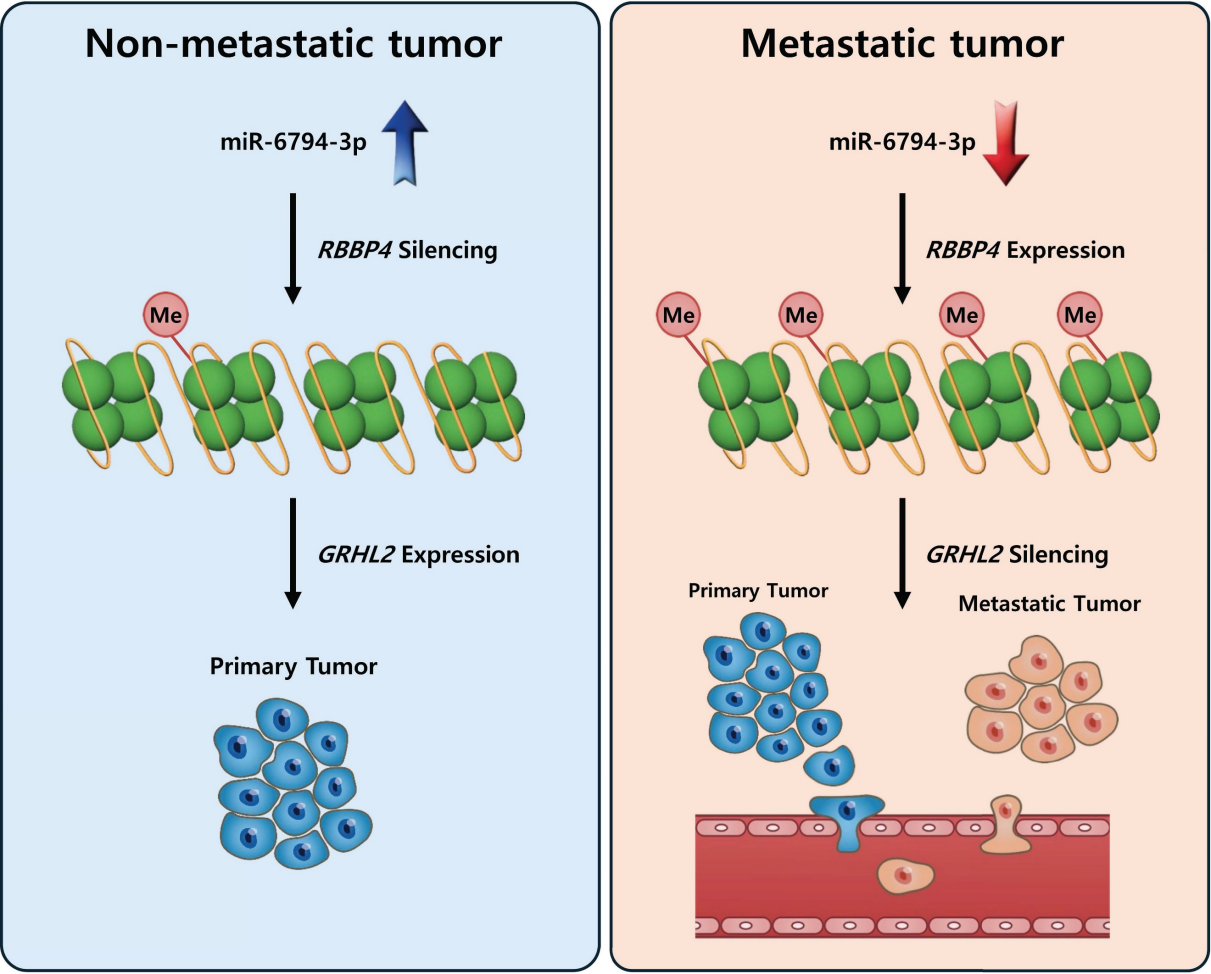
Schematic illustration of the role of miR-6794-3p in pancreatic cancer cell metastasis.

To establish the clinical value of miR-6794-3p as a potential biomarker for human pancreatic cancer, we used Kaplan-Meier analysis to examine the overall survival of patients categorized by their miR-6794-3p levels. Notably, the group of patients with low-level miR-6794-3p expression had markedly lower survival rates and shorter survival times than those with high-level miR-6794-3p expression (**Figure 1F**). However, the group with high-level miR-6794-3p expression was relatively small, so the statistical significance of this difference was relatively low. At present, there are very few publicly available datasets related to miR-6793-3p expression and metastasis in pancreatic cancer patients, and further research is needed. *In vivo*, we found that miR-6794-3p significantly suppressed pancreatic cancer cell metastasis in a mouse lung metastasis model (**Figure 7**). Our microarray analysis of metastatic and non-metastatic pancreatic tissues showed that miR-6794-3p expression was 1.5-fold lower in metastatic tissues (**Figure 1A**). For the purpose of molecular diagnosis, a 1.5-fold decrease is a somewhat small change. However, as shown in **Figure 1B**, if qPCR of biopsy specimens is used for diagnosis, a clear decrease in the level of miR-6794-3p can be seen in samples of metastatic pancreatic cancer, relative to non-metastatic samples. In addition, qPCR analysis of the expression of *RBBP4*, as a target gene of miR-6794-3p, can complement the analysis of miR-6794-3p for molecular diagnosis. Based on our collective results, we conclude that miR-6794-3p modulates tumor growth via regulation of RBBP4/GRHL2 signaling, and has considerable value as a potential biomarker of metastasis in pancreatic cancer (**Figure 8**). Our results further support the idea that miR-6794-3p could potentially be leveraged for the development of tailored treatments to combat pancreatic cancer metastasis.

## Supplementary Material

Supplementary figures and tables.
