# Supplementary material for: Identification of miR-6794-3p as a suppressor in pancreatic cancer metastasis
Source: Int J Biol Sci. 2024 Sep 30;20(13):5272–92. doi: 10.7150/ijbs.98490 (PMC11488588; doi:10.7150/ijbs.98490)
Supplement: Supplementary file 1 — Supplementary figures and tables. [file ijbsv20p5272s1.pdf]

Identification of miR-6794-3p as a suppressor in pancreatic cancer metastasis

Kim et al.,

Supplementary Figures and Tables

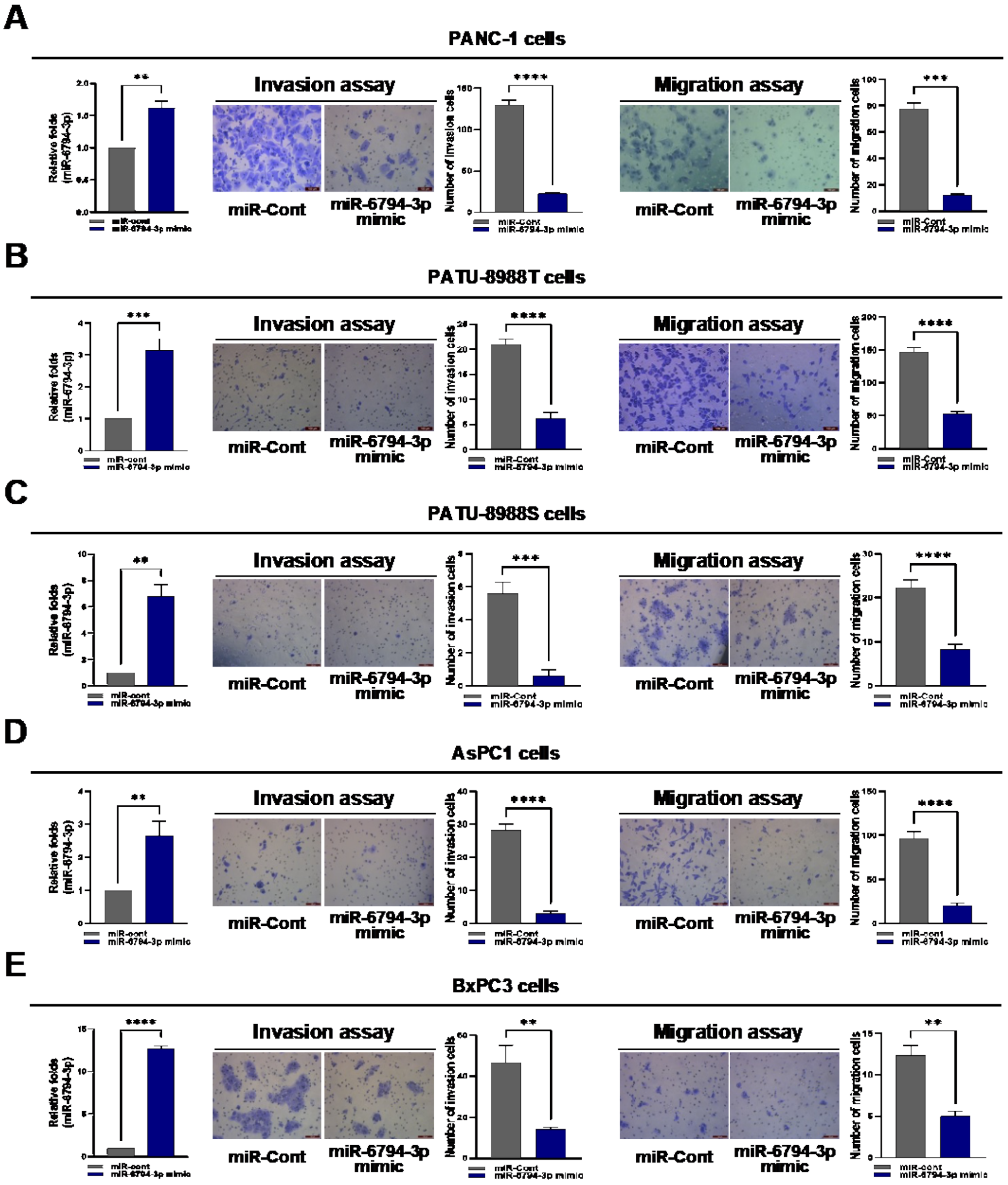

Supplementary Figure S1. Effects of miR-6794-3p on invasion and migration in various pancreatic cancer cells. **(A-E)** Invasion and migration assays on miR-Cont- or miR-6794-3p mimic-transfected pancreatic cancer cells, including PANC-1 **(A)**, PATU-8988T **(B)**, PATU-8988S **(C)**, AsPC-1 **(D)** and BxPC-3 **(E)** cells. Left panel: Relative levels of miR-6794-3p in miR-Cont- or miR-6793-3p mimic-transfected pancreatic cancer cells examined via qPCR using *U6 snRNA* as the internal control. All data are presented as mean ± SEM. ** $P < 0.01$ with unpaired *t* test, **** $P < 0.0001$ with unpaired *t* test; Mid panel: Representative images and quantification results of invasion assays in miR-Cont- or miR-6793-3p mimic- transfected pancreatic cancer cells. All data are presented as mean ± SEM. ** $P < 0.01$ with unpaired *t* test, **** $P < 0.0001$ with unpaired *t* test. Right panel: Representative images and quantification results of migration assays on miR-Cont- or miR-6793-3p mimic-transfected pancreatic cancer cells. All data are presented as mean ± SEM. ** $P < 0.01$ with unpaired *t* test, *** $P < 0.001$ with unpaired *t* test, **** $P < 0.0001$ with unpaired *t* test.

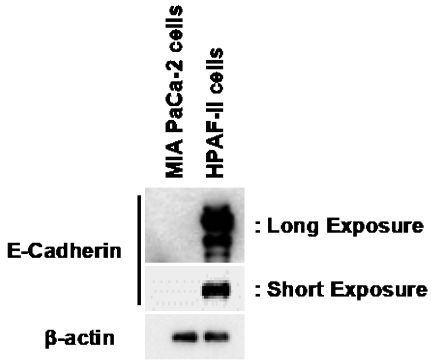

Supplementary Figure S2. Expression of E-cadherin in MIA-PaCa-2 and HPAF-II cells.

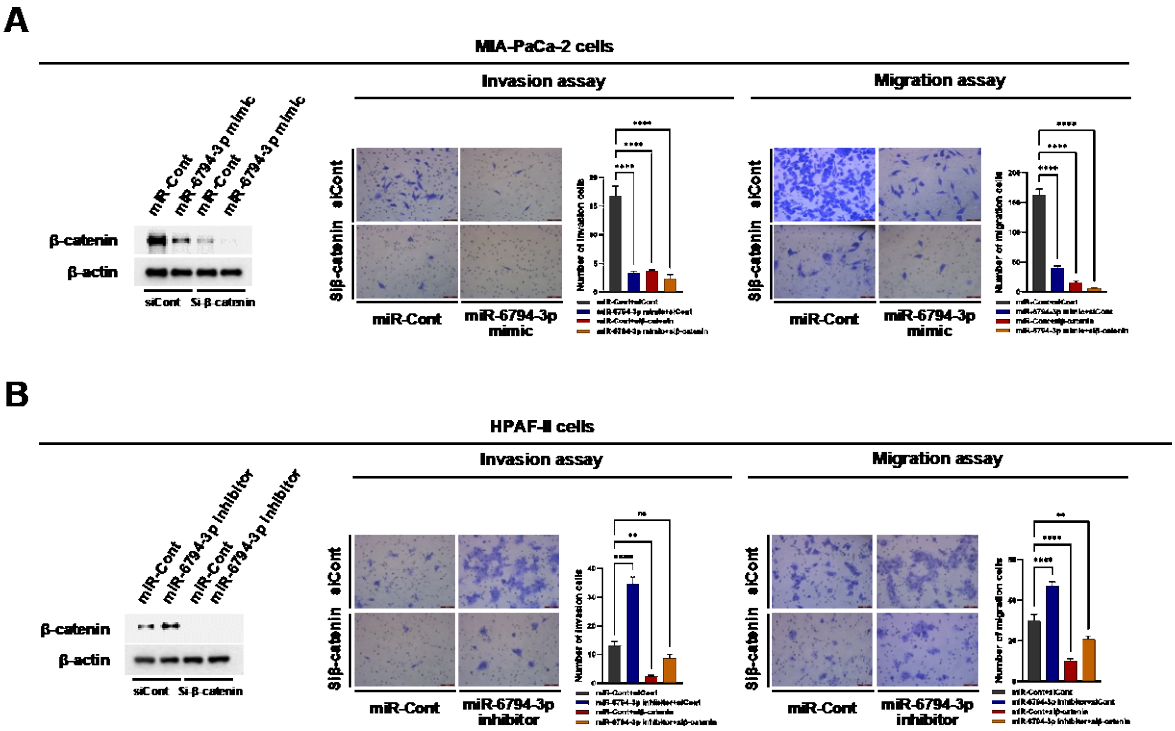

Supplementary Figure S3. Effect of β-catenin on invasion and migration of pancreatic cancer cells. **(A)** Invasion and migration assay on MIA-PaCa-2/miR-Cont cells and MIA-PaCa-2/miR-6794-3p mimic cells transfected with siCont or siβ-catenin. Left panel: The indicated proteins were analyzed via immunoblot assay. Mid panel: Representative images of invading cells and quantification of invasion. Right panel: Representative images of migrating cells and quantification of migration. All data are presented as mean ± SEM. **** $P < 0.0001$ with unpaired *t* test. **(B)** Invasion and migration assay on HPAF-II/miR-Cont cells and HPAF-II/miR-6794-3p inhibitor cells transfected with siCont or siβ-catenin. Left panel: The indicated proteins were analyzed via immunoblot assay. Mid panel: Representative images of invading cells and quantification of invasion. Right panel: Representative images of migrating cells and quantification of migration. All data are presented as mean ± SEM. ** $P < 0.01$ with unpaired *t* test, **** $P < 0.0001$ with unpaired *t* test.

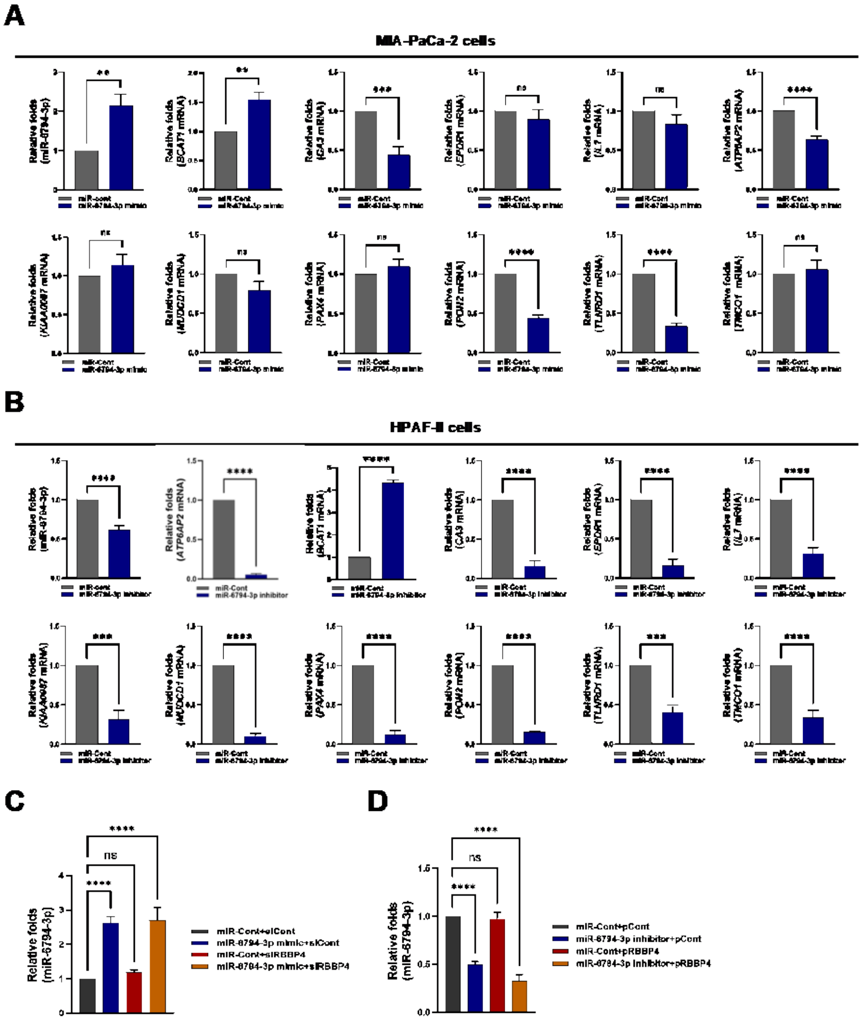

Supplementary Figure S4. Effect of miR-6794-3p on expression of *RBBP4* in pancreatic cancer cells. **(A)** Effects of miR-6794-3p expression on *RBBP4*, *ATP6AP2*, *BCAT1*, *CA3*, *EPDR1*, *IL7*, KIAA0087, *NUDCD1*, *PAX4*, *PON2*, *TLNRD1*, and *TMCO1* in miR-Cont- or miR-6794-3p mimic-transfected MIA-PaCa-2 cells examined via qPCR using *18S rRNA* as the internal control. All data are presented as mean ± SEM. ** $P < 0.01$ with unpaired *t* test, *** $P < 0.001$ with unpaired *t* test, **** $P < 0.0001$ with unpaired *t* test. Ns indicates no significance. **(B)** Effects of miR-6794-3p expression on *RBBP4*, *ATP6AP2*, *BCAT1*, *CA3*, *EPDR1*, *IL7*, KIAA0087, *NUDCD1*, *PAX4*, *PON2*, *TLNRD1*, and *TMCO1* in miR-Cont- or miR-6794-3p inhibitor-transfected HPAF-II cells examined via qPCR using *18S rRNA* as the internal control. All data are presented as mean ± SEM. *** $P < 0.001$ with unpaired *t* test, **** $P < 0.0001$ with unpaired *t* test. Ns indicates no significance. **(C)** Relative levels of miR-6794-3p in miR-Cont- or miR-6793-3p mimic- and siCont- or siRBBP4-transfected MIA-PaCa- 2 examined via qPCR using *U6 snRNA* as the internal control. All data are presented as mean ± SEM. **** $P < 0.0001$ with unpaired *t* test. Ns indicates no significance. **(D)** Relative levels of miR-6794-3p in miR-Cont- or miR-6793-3p inhibitor- and pCont- or pRBBP4- transfected HPAF-II examined via qPCR using *U6 snRNA* as the internal control. All data are presented as mean ± SEM. **** $P < 0.0001$ with unpaired *t* test. Ns indicates no significance.

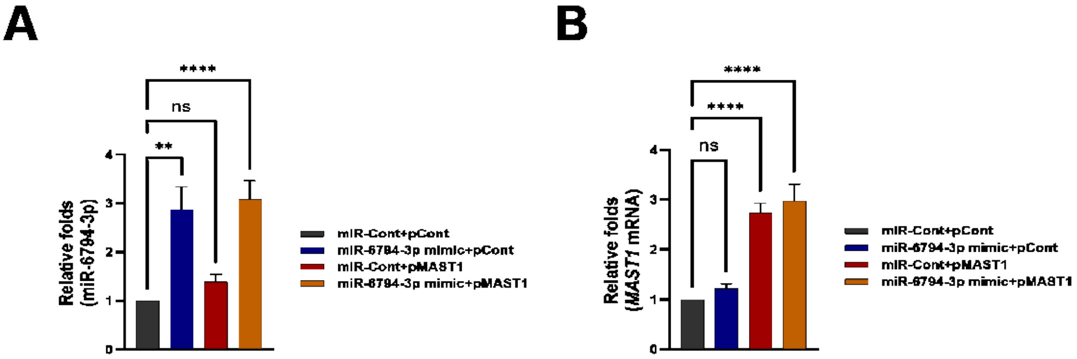

Supplementary Figure S5. Correlation between miR-6794-3p and MAST1 expression in pancreatic cancer cells. **(A)** Relative levels of miR-6794-3p in miR-Cont- or miR-6793-3p mimic- and pCont- or pMAST1-transfected MIA-PaCa-2 examined via qPCR using *U6 snRNA* as the internal control. All data are presented as mean ± SEM. ** *P* < 0.01 with unpaired *t* test, **** *P* < 0.0001 with unpaired *t* test. Ns indicates no significance. **(B)** Relative mRNA levels of MAST1 in miR-Cont- or miR-6793-3p mimic- and pCont- or pMAST1-transfected MIA-PaCa-2 examined via qPCR using *18S rRNA* as the internal control. All data are presented as mean ± SEM. **** *P* < 0.0001 with unpaired *t* test. Ns indicates no significance.

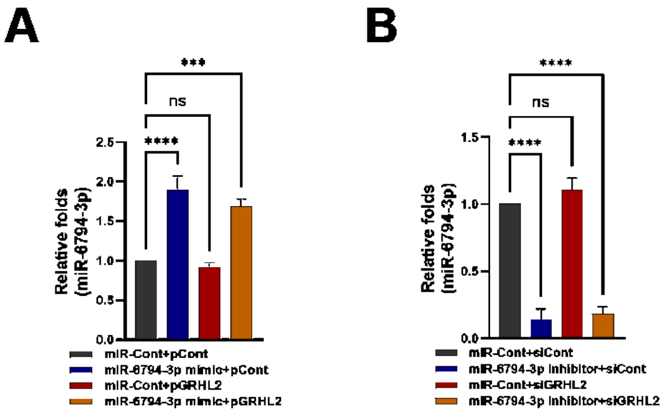

Supplementary Figure S6. Effect of GRHL2 on miR-6794-3p expression in pancreatic cancer cells. **(A)** Relative levels of miR-6794-3p in miR-Cont- or miR-6793-3p mimic- and pCont- or pGRHL2- transfected MIA-PaCa-2 examined via qPCR using *U6 snRNA* as the internal control. All data are presented as mean ± SEM. *** $P < 0.001$ with unpaired *t* test, **** $P < 0.0001$ with unpaired *t* test. Ns indicates no significance. **(B)** Relative levels of miR-6794-3p in miR-Cont- or miR-6793-3p inhibitor- and siCont- or siGRHL2-transfected HPAF-II examined via qPCR using *U6 snRNA* as the internal control. All data are presented as mean ± SEM. **** $P < 0.0001$ with unpaired *t* test. Ns indicates no significance.

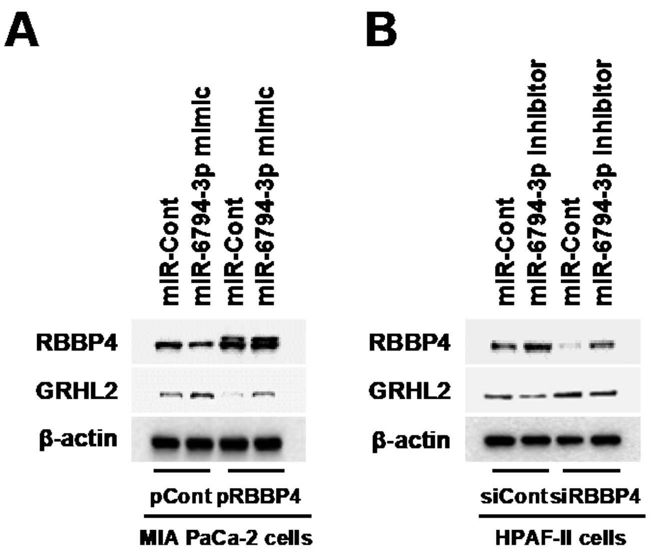

Supplementary Figure S7. Effect of miR-6794-3p/RBBP4 signaling on GRHL2 expression in pancreatic cancer cells. **(A)** Expression of GRHL2 in miR-Cont- or miR-6793-3p mimic- and pCont- or pRBBP4-transfected MIA-PaCa-2 cells. The indicated proteins were subjected to immunoblot analysis. **(B)** Expression of GRHL2 in miR-Cont- or miR-6793-3p inhibitor- and siCont- or siRBBP4-transfected HPAF-II cells. The indicated protein levels were analyzed via immunoblot assay.

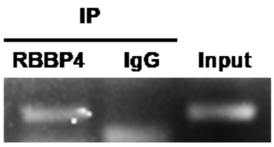

Supplementary Figure S8. ChIP assay to verify the binding of RBBP4 to GRHL2 promoter.

Supplementary Figure S9

Related to Figure 2C

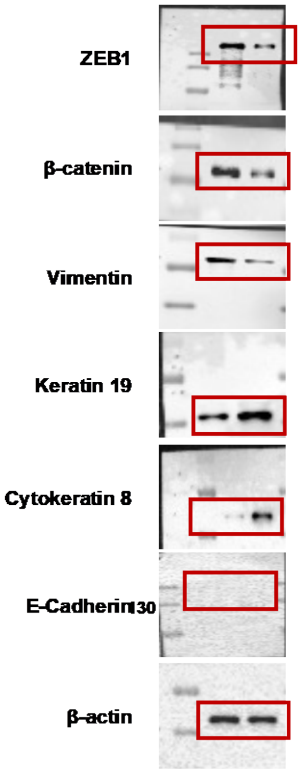

Related to Figure 2D

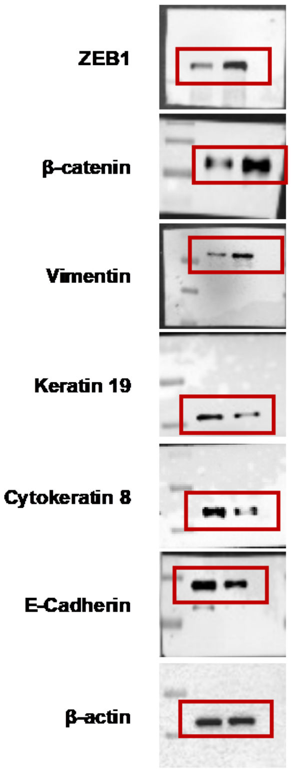

Related to Figure 3E

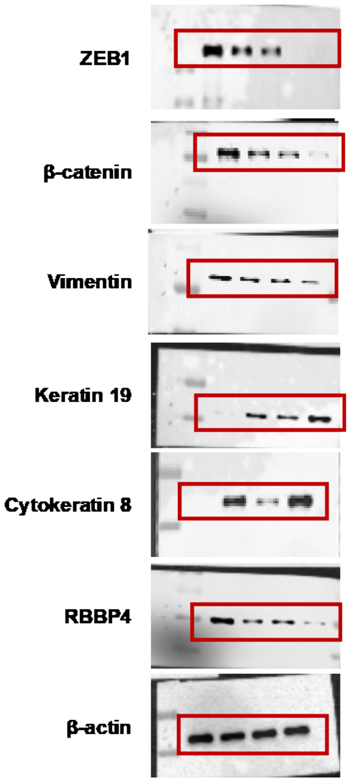

Related to Figure 3F

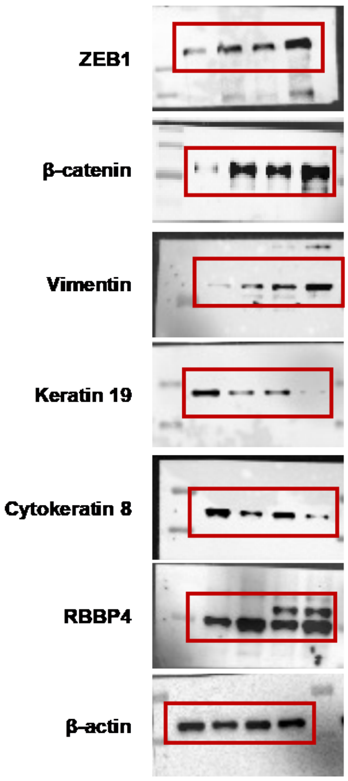

Related to Figure 5A

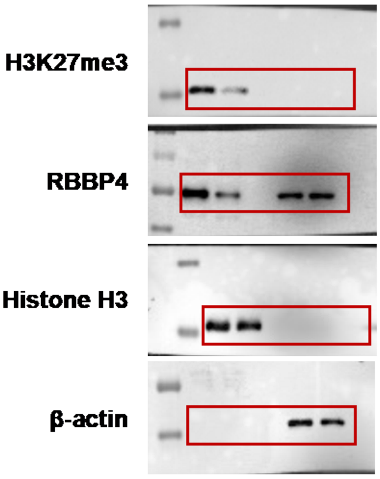

Related to Figure 5B

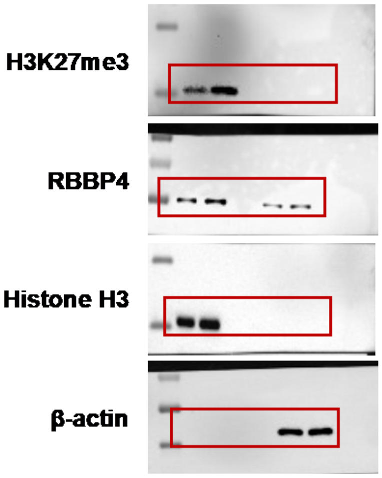

Related to Figure 5C

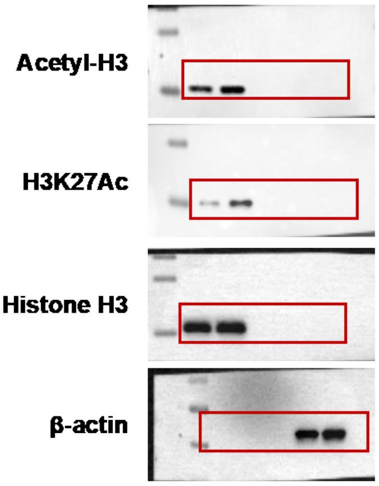

Related to Figure 5D

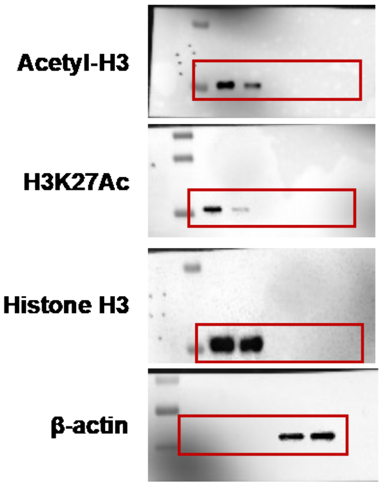

Related to Figure 5E

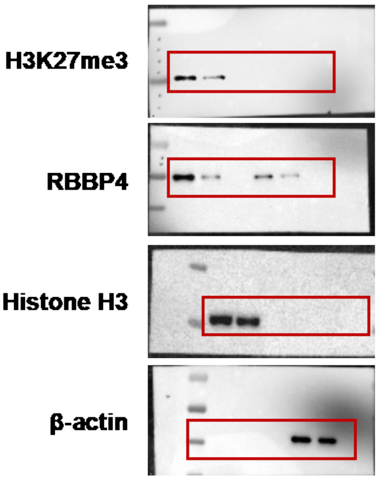

Related to Figure 5F

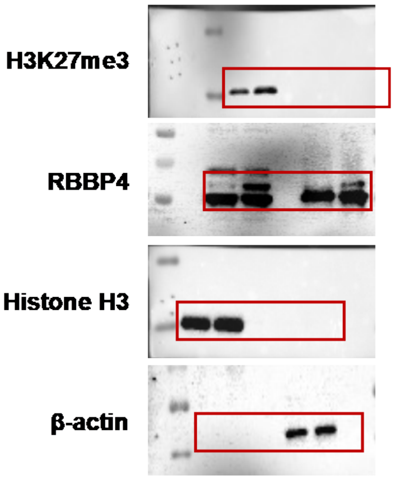

Related to Figure 5G

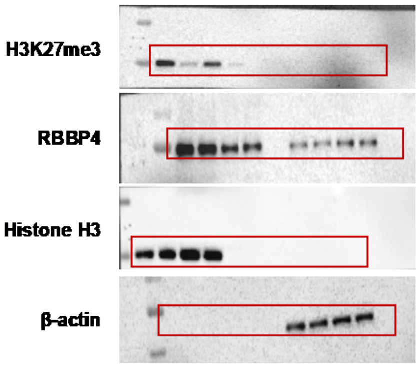

Related to Figure 5F

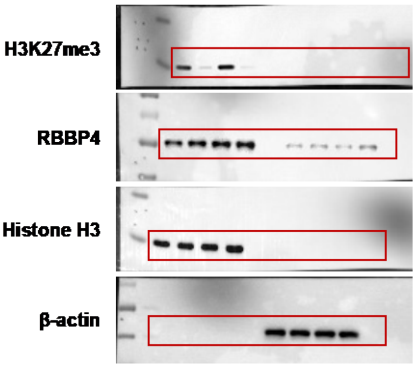

Related to Figure 6C

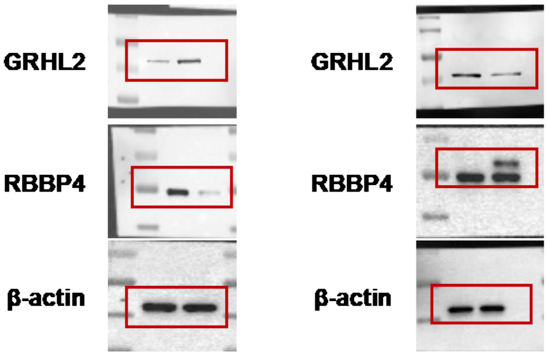

Related to Figure 6D

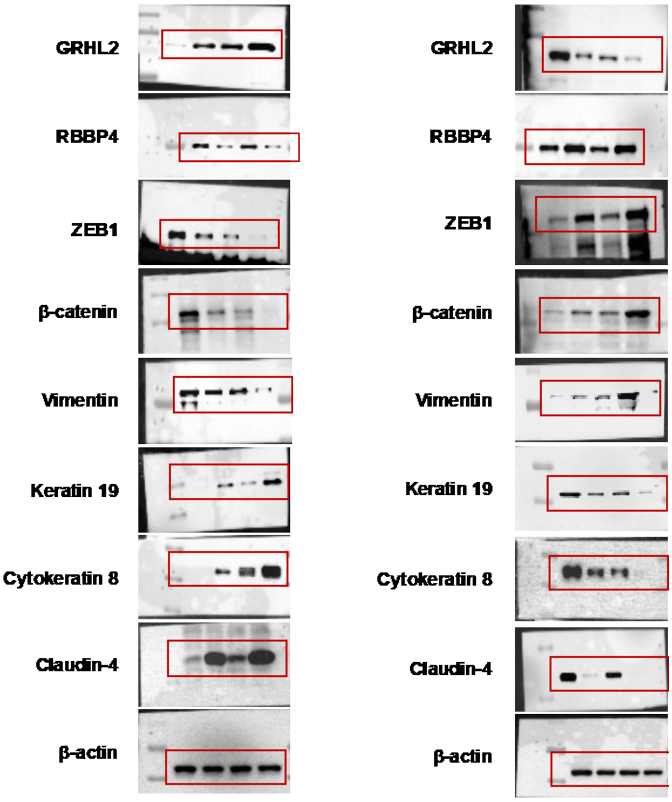

Related to Supplementary Figure S2

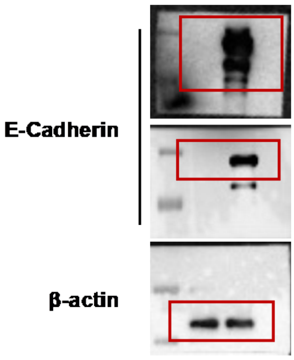

Related to Supplementary Figure S3

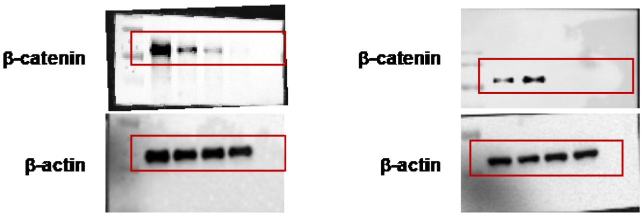

Related to Supplementary Figure S7A

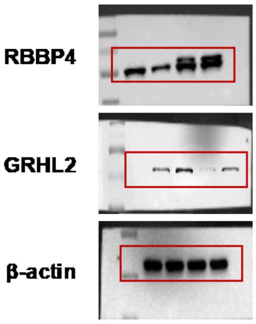

Related to Supplementary Figure S7B

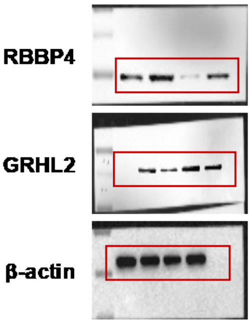

Supplementary Table 1. Sequences of specific primers used in qPCR

Gene name		Sequence
RBBP4	Forward	5'-GCT ATG GGC TTT CTT GGA-3'
	Reverse	5'-CAC AGG CAG ATG GTA TGG-3'
GRHL2	Forward	5'-CTC AGT ATG ACG TGC CCT CGC TG-3'
	Reverse	5'-GGT GGC TTC CAG GGT GTA CTG AA-3'
MAST1	Forward	5'-AAG GAG TGT CCA GTC CCG-3'
	Reverse	5'-GAC GCA CTT TAT TTA TAT GTG-3'
KRT8	Forward	5'-GCG CAC AAA GAC TGA GAT CTC-3'
	Reverse	5'-GCC TCC AGG GAA GCC CTC TG-3'
KRT19	Forward	5'-CAG CCG GAC TGA AGA ATT GAA-3'
	Reverse	5'-GTC TTC CAA GGC AGC TTT CAT-3'
CTNNB1	Forward	5'-AGA AAA GCG GCT GTT AGT CAC-3'
	Reverse	5'-CCT GTT CCC ACT CAT ACA GGA-3'
ZEB1	Forward	5'-TCC AGG AAG AAC CCT TGA ACT-3'
	Reverse	5'-CTG GGC AGT GAC TGT AGG TAT-3'
ATP6AP2	Forward	5'-TTG TTG ACG CTC TGC AAA AGT-3'
	Reverse	5'-CTG GGT TCT TCG CTT GTT TTG-3'
BCAT1	Forward	5'-TGT GTT GTT TGC CCA GTT TCT-3'
	Reverse	5'-ATT GTC CAG TCG CTC TCT TCT-3'
CA3	Forward	5'-CGT GGA TGG AGT CAA GTA TGC-3'
	Reverse	5'-GGA AAA TCT GGA ACT CGC CAT-3'
EPDR1	Forward	5'-AGT GCT CAA AGA TGA CCC TGA-3'
	Reverse	5'-TGC CAA TCC AGG TTT CAT AGG-3'
IL7	Forward	5'-TCG CAA GTT GAG GCA ATT TCT-3'
	Reverse	5'-ACT CTT TGT TGG TTG GGC TTC-3'
KIAA0087	Forward	5'-GAA TCC AGG AGC ATT TTG TC-3'
	Reverse	5'-GAG TGA ATG AAT TGG AGC A-3'
NUDCD1	Forward	5'-CTG TGG CAG AGG TAA AAC TTC-3'
	Reverse	5'-GAC AAG GTA ACC CAG GTA GAA G-3'
PAX4	Forward	5'-TGG GCA ACA GCA CCA GAA A-3'
	Reverse	5'-AAG GGC AAG GAA GGC AAA G-3'
PON2	Forward	5'-ACA TCT GGG TAG GCT GTC ATC-3'
	Reverse	5'-GGA GAA CAG ACC CAT TGT TGG-3'
TLNRD1	Forward	5'-GGG AGA GGC TGA GGA ACT C-3'
	Reverse	5'-TGG AAT TCA CTG GCG GTA AAG-3'
TMCO1	Forward	5'-GGA AAC AAT AAC AGA GTC AG-3'
	Reverse	5'-AGC TTT GCC ACC ACT CTA C-3'
MAST1	Forward	5'-AAG GAG TGT CCA GTC CCG-3'
	Reverse	5'-GAC GCA CTT TAT TTA TAT GTG-3'
VIM	Forward	5'-GCG AGG AGA GCA GGA TTT CTC-3'
	Reverse	5'-ACC AGA GGG AGT GAA TCC AGA-3'
CDH1	Forward	5'-AGC AAA GGG CTT GGA TTT TGA-3'
	Reverse	5'-CAC AAA GAT GGG GGC TTC ATT-3'
18s rRNA	Forward	5'-GTA ACC CGT TGA ACC CCA T-3'
	Reverse	5'-CCA TCC AAT CGG TAG TAG CG-3'

Supplementary Table 2. Sequences for siRNAs, miRNA mimics and miRNA inhibitors

Gene name	Sequence
RBBP4	GACAUCAGUGCCGUUCCAAdTdT
GRHL2	GAAUGAAGAGGCGAAGAUUdTdT
miR-6794-3p mimic	CAGGGGGACUGGGGGUGAGC
miR-3652 mimic	CGGCUGGAGGUGUGAGGA
miR-190a-3p mimic	UGAUAUGUUUGAUAUAUUAGGU
miR-4449 mimic	CGUCCCGGGGCUGCGCGACCGA
miR-181b-5p inhibitor	AACAUUCAUUGCUGUCGGUGGGU
miR-4454 inhibitor	GGAUCCGAGUCACGGCACCA
miR-99b-3p inhibitor	CAAGCUCGUGUCUGUGGGUCCG
miR-1261 inhibitor	AUGGAUAAGGCUUUGGCUU
miR-3065-5p inhibitor	UCAACAAAAUCACUGAUGCUGGA
miR-7975 inhibitor	AUCCUAGUCACGGCACCA
miR-1913 inhibitor	UCUGCCCCCUCCGCUGCUGCCA
miR-6794-3p inhibitor	CUCACUCUCAGUCCCUCCCU

Supplementary Table 3. Sequences of primer pairs used in methylation-specific PCR

Gene name		Sequence
Methylation	Forward	5'-TAT TGT AGT AGT TAG GTT GGG TTC G-3'
	Reverse	5'-CAT TCC GCG TTA AAA ACG AT-3'
Unmethylation	Forward	5'-GTA AGT ATT GTA GTA GTT AGG TTG GGT TTG-3'
	Reverse	5'-AAA AAC ATT CCA CAT TAA AAA CAA T-3'

Supplementary Table 4. Sequences of primer pairs used in construction of plasmids expressing RBBP4 and GRHL2

Gene name		Sequence
RBBP4	Forward	5'-GGG GTA CCA TGG CCG ACA AGG AAG C-3'
	Reverse	5'-GGA ATT CGG ACC CTT GTC CTT CTG GAT C-3'
GRHL2	Forward	5'-GGG GTA CC ATG TCA CAA GAG TCG GAC-3'
	Reverse	5'- GCT CTA GA GAT TTC CAT GAG CGT GAC C -3'

Supplementary Table 5. Sequences of primer pairs used in construction of luciferase reporter plasmids encoding the wild-type and mutant 3'-UTR of RBBP4

Gene name		Sequence
RBBP4 3`-UTR WT	Forward	5'-GCT CTA GA ATG TGT CTTBTAC TTG TTG TG-3'
	Reverse	5'-CGG GAT CC CCA TCA GCA TGG GGT TAT G-3'
RBBP4 3`-UTR 1Δ	Forward	5'- TTT AAC ACT GGT TTT GAG AC-3'
	Reverse	5'-GGT TGA GAA GAA AAA AGG GG-3'
RBBP4 3`-UTR 2Δ	Forward	5'-TAA GGA ATA GAG CCA AAT GAG-3'
	Reverse	5'-AGC CTT TAC TTA AAT GAG TC-3'
RBBP4 3`-UTR 3Δ	Forward	5'-ATC AAT AAC TTG TAT TTG-3'
	Reverse	5'-TCT CAA CTT GAC AGT CTA CC-3'

Supplementary Table 6. Sequences of nucleotide pairs used in construction of luciferase reporter plasmids encoding the 3 × GRE and 5 × GRE

Gene name		Sequence
3 × GRE	Forward	5'-GTT AAC CGG TTC TCG AGA TCT GCG ATC TAA G-3'
	Reverse	5'-CGG TTA ACC GGT TGG TAC CTA TCG ATA GAG AAA-3'
5 × GRE	Forward	5'-GTT AAC CGG TTA ACC GGT TCT CGA GAT CTG CGA TCT AAG-3'
	Reverse	5'-CGG TTA ACC GGT TAA CCG GTT GGT ACC TAT CGA TAG AGA AA-3'

Supplementary Table 7. Sequences of primer pairs used in construction of plasmids expressing exon and intron of MAST1 and sequences of nucleotide pairs used in construction of plasmid shGRHL2

Gene name		Sequence
MAST1 exon9-10	Forward	5'-GAA TTC AAC CCC GAG GAG TTC-3'
	Reverse	5'-AGA GAG ATC GTC TTG CTC AGG-3'
MAST intron 9	Forward	5'-GTG CCG GCT GGT-3'
	Reverse	5'-CTA GGG AGG GAC TGA GAG TG-3'
pre-miR-6794-3p	Forward	5'-GGG CGCAGG GGG ACT GG-3'
	Reverse	5'-AGA GAG ATC GTC TTG CTC AGG-3'
shGRHL2	Forward	5'-CTC GAG ATA ACT ACT GGA CCC AGA CGT TTT TTG AAT TCG ATA TCA TC-3'
	Reverse	5'-TAT TGA TGA CCT GGG TCT GCA CCG GTG TTTCGT CCT TTC CAC-3'
